# Synthesis of β-Cyclodextrin-Decorated Dendritic Compounds Based on EDTA Core: A New Class of PAMAM Dendrimer Analogs

**DOI:** 10.3390/pharmaceutics14112363

**Published:** 2022-11-02

**Authors:** Israel González-Méndez, Esteban Loera-Loera, Kendra Sorroza-Martínez, Mireille Vonlanthen, Fabián Cuétara-Guadarrama, María Josefa Bernad-Bernad, Ernesto Rivera, Jesús Gracia-Mora

**Affiliations:** 1 Departamento de Química Inorgánica y Nuclear, Facultad de Química, Universidad Nacional Autónoma de México, Circuito Escolar, Ciudad Universitaria, Mexico City CP 04510, Mexico; 2 Escuela de Ciencias de la Salud, Campus Coyoacán, Universidad del Valle de México, Calzada de Tlalpan 3000, Coyoacán, Mexico City CP 04910, Mexico; 3 Instituto de Investigaciones en Materiales, Universidad Nacional Autónoma de México, Circuito Exterior, Ciudad Universitaria, Mexico City CP 04510, Mexico; 4 Departamento de Farmacia, Facultad de Química, Universidad Nacional Autónoma de México, Circuito Escolar, Ciudad Universitaria, Mexico City CP 04510, Mexico

**Keywords:** EDTA dendrimer, β-cyclodextrin, click chemistry, PAMAM dendrimer, microwave assisted synthesis

## Abstract

In this work, two dendritic molecules containing an ethylenediaminetetraacetic acid (EDTA) core decorated with two and four β-cyclodextrin (βCD) units were synthesized and fully characterized. Copper(I)-catalyzed alkyne–azide cycloaddition (CuAAC) click chemistry under microwave irradiation was used to obtain the target compounds with yields up to 99%. The classical ethylenediamine (EDA) core present in PAMAM dendrimers was replaced by an EDTA core, obtaining platforms that increase the water solubility at least 80 times compared with native βCD. The synthetic methodology presented here represents a convenient alternative for the rapid and efficient construction of PAMAM analogs. These molecules are envisaged for future applications as drug carriers.

## 1. Introduction

Dendrimers are hyperbranched synthetic polymers made up of a central core, repetitive branches (dendrons) and terminal functional groups at the surface. The surface groups modulate the dendrimer physical and chemical properties such as solubility, stability at different pH values, glass transition temperature (*T*_g_), and biodegradation among others [1,2,3,4]. Dendrimers have a monodisperse molecular structure since they are synthesized through a sequence of iterative reactions that allow adequate control of different critical parameters such as size (1–100 nm), shape (compact or spherical), flexibility, and internal and surface properties [5,6,7]. The properties of dendrimers have been explored in different biological applications, for example in the development of oral and nasal drug delivery systems through encapsulation [8,9,10], the conjugation and targeting of analgesic [11], in addition to anti-inflammatory [12] and anticancer drugs [13]. Other applications are the diagnosis of diseases (contrast agents), cell transfection, tissue engineering (cell repair), globular protein mimicry (hydroxyapatite regeneration) and electrochemical sensing [14,15].

Currently, there is a wide variety of dendrimer families with great potential for biomedical and pharmaceutical applications, such as lysine [16], polypropylene imine (PPI) [17,18], polyamido amine (PAMAM) [19,20], ether-amine or ester-amine [21,22], and phosphorus dendrimers [23,24]. PAMAM dendrimers are the most studied, characterized and used dendrimers in biomedical and pharmaceutical areas [25,26].

Two general methodologies are employed for the synthesis of dendrimers: in the divergent method the synthesis starts from the core and the branches are grown in subsequent steps, while in the convergent method each of the big branches (dendrons) are synthesized individually and then joined together at the core [27,28]. The PAMAM dendrimer core is composed of molecular linear chains of primary amines such as ethylenediamine (EDA) (four-core multiplicity), ammonia (three-core multiplicity) or cystamine (four-core multiplicity) [29]. The classical synthesis of the branches in PAMAM dendrimers is carried out by “iterative” Michael addition reactions of the amino terminal groups with methyl acrylate derivative. This followed by the amidation of the ester group, conducted in turn by adding an excess of EDA, which thus enables exponential growth with control of the terminal functionalities [29,30,31].

PAMAM dendrimers present some limitations in terms of their synthesis and purification. In the divergent method, incomplete and secondary undesired reactions can lead to incomplete or faulty dendrimers. For instance, partial Michael addition reactions give rise to structures with only one branch; unwanted intra- and intermolecular cyclization reactions, where the amidation is carried out between two branches of the same or different dendrons, respectively; retro-Michael reactions, in which a branch degrades generating free amines; and the hydrolysis of acrylate esters, giving rise to a nonreactive carboxylic acid [32,33,34]. In contrast, in the convergent synthesis, purer and more uniform PAMAM dendrimers can be obtained. However, this methodology has the disadvantage of presenting steric hindrance in high-generation dendrimers, leading to low yields. It should be noted that this type of synthesis is not suitable for large scale production, since large numbers of impurities with similar composition and physicochemical properties are obtained, making purification difficult in this process [32,35]. This is of great relevance, since it has been reported that impurities have a negative impact on the targeted administration of anticancer PAMAM dendrimers in preclinical trials [36,37,38].

Another important aspect during the design and synthesis of dendrimers is the quantity and nature of the functional groups found at the periphery, since they are largely related to the toxicity of these systems. On the one hand, dendrimers with cationic groups on their surface are expected to be highly toxic; moreover, dendrimers with anionic or neutral groups have little or no toxicity. Therefore, surface structural modifications introducing different functional groups can make dendrimers biocompatible for biological applications [39,40]. Surface modification strategies can be originated from covalent or noncovalent bond formation from lipids (fatty acids), fluorinated compounds (perfluoroalkyl acids), proteins or peptides (lysine and arginine), polymers (polyethylene glycol), nanoparticles (carbon nanotubes, gold nanoparticles, etc.) and saccharides (mannose, lactose, etc.) [41,42].

Cyclodextrins (CD) are the most used saccharides for surface modifications of dendrimers due to their broad availability and low cost. Recently, surface engineering with β-cyclodextrin (βCD) of G0 and G1 PAMAM dendrimers has been reported as a promising strategy for drug delivery via nanocarriers. The βCD cavity possesses the ideal size to form inclusion complexes with small- to medium-sized drugs, endowing PAMAM dendrimers with the ability to transport more than one drug molecule per unit. Furthermore, this structural modification improves the water solubility and nontoxic properties of PAMAM dendrimers [43,44]. This strategy improves the efficacy, stability, safety, bioavailability (by increasing drug solubility) and drug loading capacity in lipids, proteins and peptides for oral and parenteral administration routes [45,46,47].

**Figure 1:**
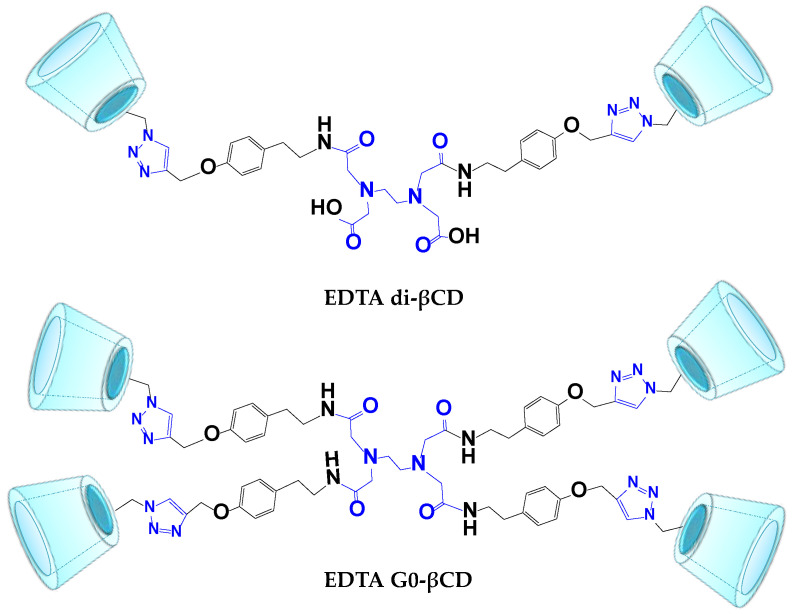
Schematic representation of the dendritic **EDTA di-βCD** and **EDTA G0-βCD** dendrimer molecules.

In recent years, nitrogen-containing dendrimers have been used as peptidomimetic agents, with promising applications for the delivery of anticancer drugs [48]. In this regard, nitrogen-containing dendrimers with an ethylenediaminetetraacetic acid (EDTA) core have shown important properties for drug delivery applications [49,50]. For those reasons and due to the drawbacks described above with the use of PAMAM dendrimers, in this work we present the design, synthesis and full characterization of new dendritic and G0-dendrimer PAMAM analogs with an EDTA core, functionalized with two and four βCD units on the periphery, EDTA di-βCD and EDTA G0-βCD compounds, respectively (see Figure 1). We have used two powerful methodologies: click chemistry through copper(I)-catalyzed alkyne–azide cycloaddition (CuAAC) in standard conditions, and microwave irradiation. This synthetic approach enabled the obtention of EDTA di-βCD dendritic molecule in high yields and short reaction times. Having two empty βCD on the periphery, EDTA di-βCD has the capacity to transport two guest molecules. Furthermore, the remaining COOH functional groups can be used for later vectorization with different functionalities. The EDTA G0-βCD dendrimer has potential increased drug-loading capacities due to the presence of four βCD units in the periphery. The structural design of EDTA core βCD dendrimers presented here offers an alternative to the classic EDA core PAMAM dendrimers, with potential applications as new drug carriers.

## 2. Results and Discussion

### 2.1. Synthesis

**Scheme 1:**
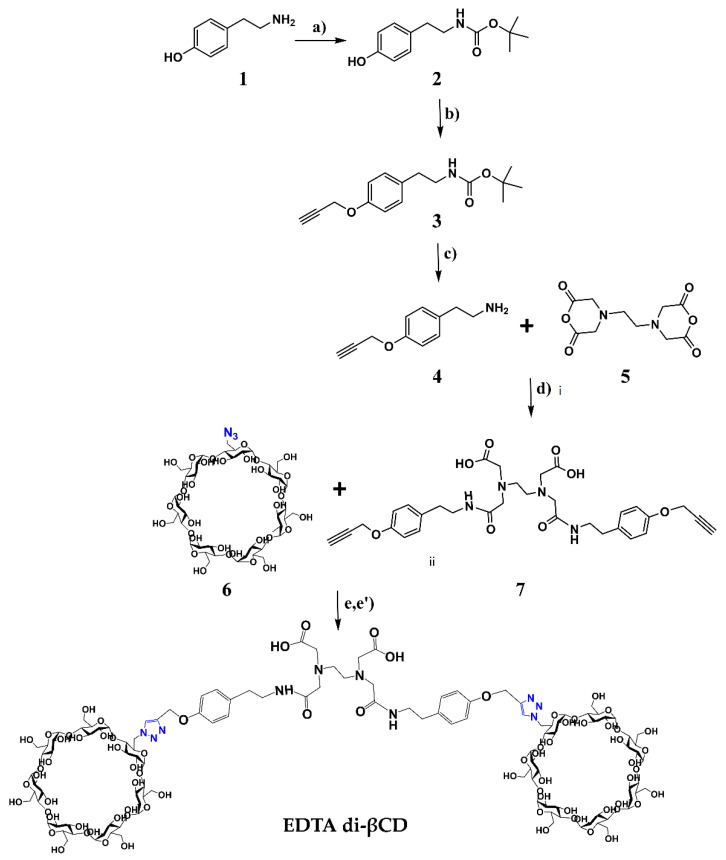
Synthesis of dendritic compound **EDTA di-βCD**. Conditions: (a) Boc_2_O/THF 0 °C, (b) K_2_CO_3_, propargyl bromide/DMF anhydrous, 80 °C, 24 h. (c) TFA/CH_2_Cl_2_, 0 °C, 3 h. (d) i. DIPEA/DMF 0 °C, ii. 2.2 eq of H_2_N(CH_2_)_2_C_6_H_4_-O-CH_2_C≡CH, (e) mN_3_-βCD, CuSO_4_•5H_2_O, H_2_Asc, DMSO:H_2_O (9:1), 80 °C, 12 h. (e’) mN_3_-βCD, CuSO_4_•5H_2_O, H_2_Asc, DMSO:H_2_O (9:1), 60 W, 90 °C, 30 min.

**Scheme 2:**
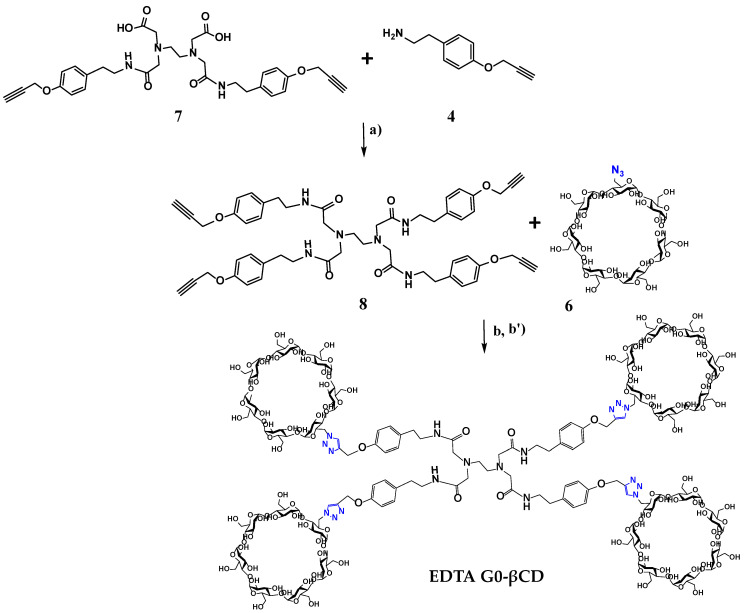
Synthesis of **EDTA G0-βCD** dendrimer. Conditions: (a) EDC·HCl, HOBt, 2.2 eq H_2_N(CH_2_)_2_C_6_H_4_-O-CH_2_C≡CH/DMF anhydrous, 24 h. (b) mN_3_-βCD, CuSO_4_•5H_2_O, H_2_Asc, DMSO:H_2_O (9:1), 80 °C, 12 h. (b’) mN_3_-βCD, CuSO_4_•5H_2_O, H_2_Asc, DMSO:H_2_O (9:1), 60 W, 90 °C, 30 min.

The synthetic pathway of **EDTA di-βCD** and **EDTA G0-βCD** dendritic molecules is shown in Scheme 1 and Scheme 2. The synthesis started with the functionalization of the EDTA core with propargyl tyramine derivative **4**. Tyramine (**1**) was selectively protected by the slow addition of di-*tert*-butyl dicarbonate (Boc_2_O) under controlled temperatures in anhydrous tetrahydrofuran (THF) [51,52], giving *N*-Boc tyramine **2** in a 98% yield. Intermediate **2** was used for Williamson etherification in the presence of propargyl bromide in basic conditions and anhydrous *N,N*-dimethylformamide (DMF) in order to obtain propargylated intermediate **3** in a 93% yield [53]. Subsequently, compound **3** was deprotected in 1:1 dichloromethane:trifluoro acetic acid (CH_2_Cl_2_:TFA) mixture at low temperatures, leading to compound **4** [54]. An acid–base extraction was performed after the deprotection reaction was completed. Product **4** in the –NH_3_^+^ protonated form was present in the aqueous phase. Extraction of compound **4** in the organic phase was done after adjusting the pH value to 12. In this way, the exposition time of product **4** in a strongly acidic medium was greatly reduced, making it possible to recover the desired compound **4** in a 91% yield without the need for further purification.

The design of the dendrimer molecules presented in this work focused on the replacement of the classical ethylenediamine core, present in PAMAM dendrimers, by an EDTA core. Base-catalyzed nucleophilic addition using *N*,*N*-Diisopropylethylamine (DIPEA) between 2 equivalents of propargyl tyramine **4** and EDTA dianhydride **5** in DMF and subsequent recrystallization from MeOH gave disubstituted EDTA alkyne **7** in a 99% yield [55]. Following a methodology reported by our group (see Scheme 2), C–N coupling between compound **7** and a slight excess of **4** was performed using *N*-(3-dimethylaminopropyl)-*N*-ethylcarbodiimide hydrochloride (EDC·HCl) and 1-hydroxybenzotriazole hydrate (HOBt) giving tetrasubstituted EDTAG0-alkyne **8** in a 98% yield after recrystallization from methanol (MeOH) [43].

β-CD was monofunctionalized at the primary face with an azide group, according to a previously reported procedure, to afford the 6-*O*-Monoazido-β-cyclodextrin (**6**) in a quantitative yield [56,57,58]. Finally, to obtain the dendritic molecule **EDTA di-βCD** and β-cyclodextrin-decorated dendrimer **EDTA G0-βCD**, the CuAAC reaction between azide **6** and compounds **7** and **8**, respectively, was performed under thermal heating for 12 h according to our reported methodology [53]. A Cu(I) source is generated in situ by reacting copper sulfate pentahydrate (CuSO_4_•5H_2_O) with ascorbic acid (H_2_Asc) as the reducing agent in a dimethylsulfoxide:water (DMSO:H_2_O) solvent mixture. A good solubility of **6** and alkynes (**7** and **8**) was observed in this mixture of solvents. An excess of **6** was added to the reaction mixture to ensure complete functionalization of the alkyne groups. As an alternative to the thermal heating, the CuAAC reaction was performed under microwave activation keeping the same proportions of reactants. A ramp time of 3 min to reach a temperature of 90 °C was implemented on the microwave apparatus, and the click reaction was performed under constant irradiation of 60 W for 30 min. Compared to the reaction time of 12 h described in the classical methodology, the use of a microwave leads to the same reaction yield in a shorter time. Independently of the synthetic conditions (thermal or microwave), the final dendritic **EDTA di-βCD** and **EDTA G0-βCD** dendrimer were purified by size exclusion chromatography, using water as an eluent, and were obtained in high purity in ≥98% yields.

### 2.2. Characterization

Key NMR signal changes of intermediate and final products were tracked by NMR spectroscopy in DMSO-*d_6_*. The functional group modifications on each synthetic step were followed by IR spectroscopy. The intermediate and final compounds molecular weights were corroborated by mass spectrometry using DART, ESI or MALDI techniques, depending on the size of the molecule.

The tyramine amine protection reaction was evidenced by the appearance of a singlet signal at 1.37 ppm with integration of nine protons in the ^1^H-NMR spectrum corresponding to Boc’s *tert*-butyl protons of compound **2**. The signal at 8.88 pm of phenol group was preserved in the ^1^H-NMR spectrum of compound **2** (see Figure S1 in SI). In the ^13^C-NMR spectrum of compound **2**, the signals at 78.2 and 29.1 ppm correspond to the quaternary and methyl groups carbon atoms, respectively (see Figure S2 in SI).

In the ^1^H-NMR spectrum of compound **3**, a doublet and singlet signals appeared at 4.76 and 3.53 ppm, corresponding to the methylene group and the terminal alkyne group protons, respectively (see Figure S5 in SI). Furthermore, the phenolic proton signal at 8.88 pm present in compound **2** disappeared due to derivatization of compound **3**. The ^13^C-NMR spectrum of compound **3** showed the signals corresponding to the alkyne group carbons, which appeared at 78.8 and 78.2 ppm for the quaternary and tertiary carbons, respectively (see Figure S6 in SI). The structure of the intermediate **2** and **3** was confirmed by mass spectrometry using the DART technique (see Figures S4 and S8, SI), where the molecular ions appeared at 238 and 276 *m*/*z*, respectively, which corresponded to the expected molecular weights.

After the deprotection reaction of intermediate **3**, a broad signal due to the amine protons of compound **4** appeared at 2.19 ppm in the ^1^H-NMR spectrum (see Figure S9 in SI). Furthermore, the disappearance of the Boc methyl protons was observed. In the ^13^C-NMR spectrum, the signals corresponding to the alkyne carbon atoms were preserved (see Figure S10 in SI). The signals of the amide protons of compounds **7** and **8** appeared at 8.18 and 7.96 ppm, respectively, in the ^1^H-NMR spectra. The aromatic protons of the *para*-substituted ring of compounds **7** and **8**, appeared at 7.21 and 6.87 ppm, corresponding to Hd and Hf, respectively. The terminal alkyne proton signal appeared between 4.77 and 3.54 ppm for Hg and Hh in both molecules. The following signals were assigned to the EDTA cores. The signals corresponding to the Hc protons of the EDTA core of intermediate **7** appeared to overlap with H_2_O signals at 3.36 ppm in the ^1^H-NMR spectrum. The nonequivalent H-b,b’ protons appeared at 2.97 ppm, while a multiplet signal assigned to Hd appeared at 2.86–2.89 ppm and to the Ha protons at 2.81 ppm. The ^1^H-NMR spectrum of intermediate **8** showed a pattern of signals equivalent to that described for **7** (see Figures S13 and S17 in SI).

**Figure 2:**
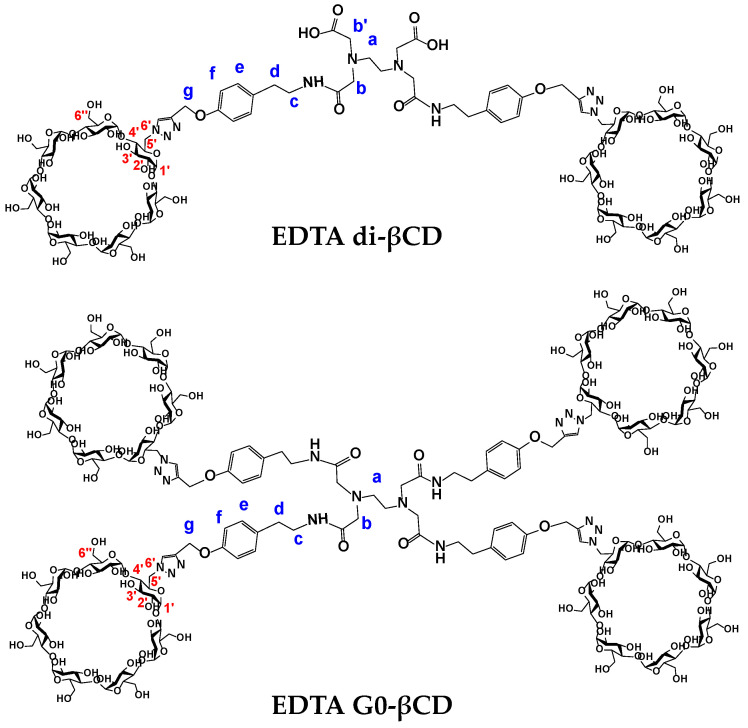
Assignation of the protons in ^1^H-NMR spectra for dendritic **EDTA di-βCD** and **EDTA G0-βCD** dendrimer.

**Figure 3:**
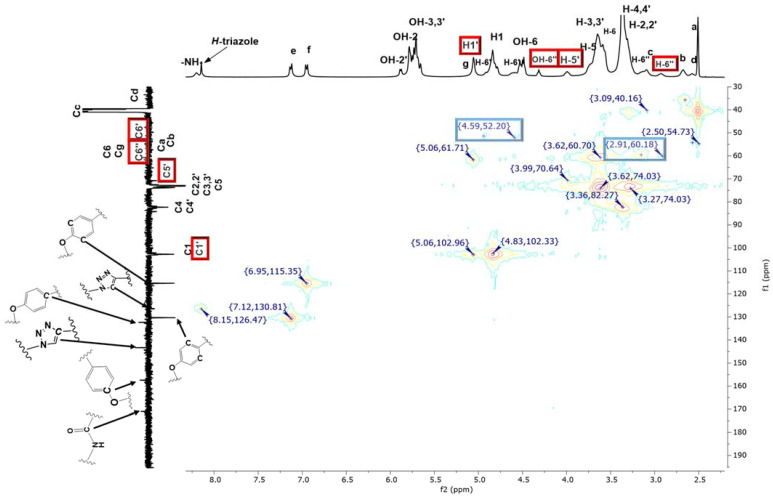
HMQC-NMR spectra of EDTA G0-βCD dendrimer.

The full characterization of the new dendritic **EDTA di-βCD** and **EDTA G0-βCD** dendrimer was performed using NMR techniques in DMSO-*d*_6_ (^1^H-,^13^C-NMR and 2D NMR HMQC and COSY, see Figures S21–S24 and S27–S29, respectively in SI). We focused on assigning the classical proton signals for the 1,4-disubstituted triazole group and all the peripheral β-CD units (see Figure 2). As can be seen in Figure 3, the H-1′, H-5′ and particularly H-6′ signals of the functionalized glucopyranose ring in β-CD appeared at lower fields than those of their respective nonfunctionalized subunits, due to the change in their chemical environment due to the presence of the triazole group. The diastereotopic H-6″ protons of the –CH_2_–OH fragment, contiguous to the functionalized glucopyranose ring, were identified at 3.14 and 2.91 ppm using HMQC 2D NMR (Figure 3). Additionally, the H-6″ proton and the adjacent OH-6″ proton, located on the primary face (*ca*. 4.32 ppm) appeared significantly upfield-shifted compared to their respective native H-6 and OH-6 counterparts, as a result of the change in chemical environment due to neighboring substitution. These features were common to both dendritic compounds, in which the β-CD cavities behave similarly. In this regard, in a recent study published by our research group, a similar behavior was observed for the protons of the substituted glucopyranose ring of PAMAM G0-βCD dendrimer and the effect that these protons exert on the proton signals of the neighboring nonfunctionalized glucopyranose groups [43].

Finally, the proposed structure of the dendritic compound **EDTA di-βCD** and the **EDTA G0-βCD** dendrimer was corroborated by ESI and MALDI-TOF mass spectrometry (Figures S26–S31 in SI), where the molecular ions appeared at 1500 and 5629 *m*/*z*, respectively, corresponding to the expected molecular weights of the final compounds with an additional potassium ion.

### 2.3. Determination of Water Solubility for EDTA di-βCD and EDTA G0-βCD

Among the specific physical properties of dendrimers, high solubility in different solvents, especially in water, makes them suitable candidates when considering biological applications compared with linear polymers. In most cases, the water solubility of the nitrogen-containing dendrimers mainly depends on the reactivity of the peripheral terminal groups [48]. Water solubility determination of EDTA di-βCD dendritic and EDTA G0-βCD dendrimer molecules was carried out according to a previously reported methodology [59]. It was found that, for both molecules, the solubility in water was >1.56 g/mL. These results demonstrate that the molecules based in EDTA-βCD are at least 80 times more soluble than the native βCD (18.5 mg/mL), and even more soluble than other commercial βCD derivatives, such as sulfobutylether-βCD (>500 mg/mL), O-methyl-βCD (>500 mg/mL) and 2-hydroxypropyl-βCD (>600 mg/mL) [58]. The remarkable solubility in water of the compounds reported in this work was conferred by the conjugation of βCD units on the periphery.

## 3. Materials and Methods

### 3.1. General Notes

Azide **6** was synthesized according to a previously reported procedure [55,56,57]. All the starting materials were purchased from Merck Sigma-Aldrich, Mexico, and used without any further purification. Bio-Gel P-6 medium was purchased from BIO-RAD. Deuterated dimethyl sulfoxide (DMSO-*d_6_*) with an isotopic purity of 99.9% was obtained from Cambridge Isotope Laboratories, Inc. Tetramethylsilane (TMS), an internal NMR reference, was purchased from Merck Sigma-Aldrich, Mexico. ^1^H and ^13^C-DEPTQ NMR, as well as 2D HMQC, COSY and NOESY experiments, were performed at 298 K on a Bruker Avance 400 MHz instrument. Chemical shifts (δ) are reported in ppm and coupling constants (*J*) in Hz. Multiplicities are reported using the following abbreviations: *s* = singlet, *d* = doublet, *t* = triplet, *br* = broad and *m* = multiplet. Proton and carbon signal assignments are indicated on the spectra reported in SI. Infrared spectra were recorded on a Nicolet FT-5SX spectrophotometer. DART, ESI and MALDI-TOF mass measurements were performed on a JEOL JMS-AX505-HA instrument (Peabody, MA, USA) and on a Bruker Daltonics Flex Analysis instrument (Bruker, Beerlika, MA, USA), respectively. 2,5-Dihydroxybenzoic acid (DHB) was used as a matrix for MALDI-TOF.

### 3.2. Synthetic Procedures

#### 3.2.1. Synthesis of tert-butyl (4-hydroxyphenethyl)carbamate (**2**)

A solution of Boc_2_O (9.55 g, 43.7 mmol) in THF (5 mL) was added dropwise to a suspension of tyramine (**1**) (5.00 g, 36.4 mmol) in THF (25 mL) at 0 °C over a period of 20 min, under vigorous stirring. The reaction mixture was stirred at 0 °C for an additional 20 min. Then, it was allowed to slowly warm to room temperature and stirred for 4 h. Once the reaction was completed (TLC-monitored), the solvent was removed under reduced pressure. The residue was dissolved in ethyl acetate and the organic phase was washed once with a saturated solution of sodium bicarbonate (NaHCO_3_) and twice with brine, dried over anhydrous sodium sulfate (Na_2_SO_4_) and the solvent was removed under reduced pressure. The crude product was purified by flash chromatography (silica gel, CH_2_Cl_2_ as eluent). The *N*-Boc-protected tyramine **2** was obtained as colorless crystals (8.47 g, 35.7 mmol, 98%). R_f_ = 0.5 (CH_2_Cl_2_). ^1^H NMR (400 MHz, DMSO-*d*_6_, δ ppm): 9.16 (s, 1H, Ph-OH), 6.98 (d, *J* = 8.3 Hz, 2H, Hd), 6.82 (m, 1H, -NH-), 6.68 (d, *J* = 8.3 Hz, 2H, He), 3.05 (m, 2H, Hb), 2.56 (t, *J* = 6.9 Hz, 2H, Hc), 1.37 (s, 9H, Ha); ^13^C-DEPTQ NMR (101 MHz, DMSO-*d*_6_, δ ppm): 156.38, 156.30, 130.27, 130.24, 115.86, 78.23, 42.70, 36.52, 29.08; IR (ATR, cm^−1^): 3370 O-H (st), 2981, 2939 CH_2_ (st), 2864 CH_3_ (st), 1892 aromatic (st); MS (DART^+^) *m*/*z*: [M + H]^+^ calculated for C_13_H_19_NO_3_ 238.30, found 238.

#### 3.2.2. Synthesis of tert-butyl (4-(prop-2-yn-1-yloxy)phenyl)carbamate (**3**)

The synthesis of alkyne intermediate was carried out according to a previously reported procedure, with some modifications [43,52]. The *tert*-Butyl (4-hydroxyphenethyl)carbamate (**2**) (13.00 g, 54.8 mmol) was dissolved in DMF (250 mL) and potassium carbonate (K_2_CO_3_) (10.60 g, 76.7 mmol) was added. The reaction mixture was heated at 80 °C for 1 h. Afterwards, propargyl bromide (7.9 mL, 71.2 mmol) was added, and the resulting mixture was refluxed for 24 h. Then, the reaction mixture was cooled to room temperature and filtered, and the filtrate was evaporated under reduced pressure. The resulting brown oil was dissolved in CH_2_Cl_2_ (150 mL), and the solution was extracted with sodium hydroxide (NaOH 1 M, 200 mL), followed by water (3 × 50 mL). The organic phase was dried over anhydrous Na_2_SO_4_ and the solvent was evaporated under reduced pressure. The crude product was purified by flash chromatography (silica gel, CH_2_Cl_2_ as eluent) to afford compound **3** as a yellow liquid (12.60 g, 50.95 mmol, 93% yield). R_f_ = 0.65 (CH_2_Cl_2_). ^1^H NMR (400 MHz, DMSO-*d*_6_, δ ppm): 7.13 (d, *J* = 8.2 Hz, 2H, Hd), 6.92 (d, *J* = 8.2 Hz, 2H, He), 6.85 (m, 1H, -NH-), 4.76 (d, 2H, Hf), 3.53 (t, 1H, Hg), 3.14 (m, 2H, Hb), 2.66 (m, 2H, Hc), 1.38 (s, 9H, Ha); ^13^C-DEPTQ NMR (101 MHz, DMSO-*d*_6_, δ ppm): 156.41, 156.32, 132.94, 130.34, 115.46, 80.19, 78.78, 78.26, 56.12, 42.52, 35.42, 29.06; IR (ATR, cm^−1^): 3357.65 N-H (st), 3292 C-H alkyne (st), 2121 C≡C (st), 2977, 2932 CH_2_ (st), 2868 CH_3_ (st),1884 aromatic (st); MS (DART^+^) *m*/*z*: [M + H]^+^ calculated for C_16_H_21_NO_3_ 276.35, found 276.

#### 3.2.3. Synthesis of 2-(4-(prop-2-yn-1-yloxy)phenyl)ethan-1-amine (**4**)

TFA (5 mL, 65.30 mmol) was added to a solution of **3** (3.00 g, 10.89 mmol) in CH_2_Cl_2_ (5 mL) at 0 °C, in and the reaction mixture was stirred for 30 min. After this time, the ice bath was removed, and the mixture was stirred for 2.5 h. After the deprotection reaction was completed (TLC-monitored), water (150 mL) was added to the reaction mixture and the organic phase was separated. The aqueous phase was treated with a 5 M of NaOH solution until reaching pH = 12 and extracted with CH_2_Cl_2_ (3 × 50 mL). The organic phase was dried over anhydrous Na_2_SO_4_ and then the solvent was evaporated to obtain compound **4** as a yellow liquid without further purification (1.73 g, 9.87 mmol, 91%). Rf = 0.3 (CH_2_Cl_2_/MeOH (9:1 *v*:*v*)). ^1^H NMR (400 MHz, DMSO-*d*_6_, δ ppm): 7.14 (d, *J* = 8.2 Hz, 2H, Hc), 6.91 (d, *J* = 8.2 Hz, 2H, Hd), 4.75 (d, 2H, He), 3.55 (t, 1H, Hf), 2.76 (m, 2H, Ha), 2.60 (m, 2H, Hb), 2.19 (br, 2H, -NH_2_); ^13^C-DEPTQ NMR (101 MHz, DMSO-*d*_6_, δ ppm): 156.23, 133.94, 130.34, 115.43, 80.21, 78.85, 56.13, 44.65; IR (ATR, cm^−1^): 3356 N-H (st), 3284 C-H alkyne (st), 2926, 2863 CH_2_ (st), 2119 C≡C (st), 1886 aromatic (st); MS (DART^+^) *m*/*z*: [M + H]^+^ calculated for C_11_H_13_NO 176.23, found 176.

#### 3.2.4. Synthesis of Disubstituted EDTA Alkyne (**7**)

DIPEA (0.75 mL) was added to a suspension of EDTA dianhydride 5 (0.50 g, 1.95 mmol) in DMF (5 mL) at 0 °C and the reaction mixture was stirred for 20 min. Then, compound 4 (0.752 g, 4.29 mmol) was added dropwise and the reaction mixture was stirred at 0 °C for 10 min and at room temperature for 12 h. The solvent was removed under reduced pressure. The solid was obtained by addition of cold MeOH. The crude product was purified by recrystallization from methanol to obtain compound 7 as a white solid (1.17 g, 1.93 mmol, 99%). ^1^H NMR (400 MHz, DMSO-d_6_, δ ppm): 7.96 (s, 2H, -CONH-), 7.20 (d, *J* = 8.21 Hz, 4H, He), 6.95 (d, *J* = 8.22 Hz, 4H, Hf), 4.77 (d, 4H, Hg), 3.56 (t, 2H, Hh), 3.36 (m, 8H, Hc overlaped with H_2_O), 3.01–2.97 (m, 4H, Hb, b’), 2.86 (s, 4H, Hd), 2.81 (m, 4H, Ha); ^13^C-DEPTQ NMR (101 MHz, DMSO-d_6_, δ ppm): 173.68, 156.79, 131.01, 130.50, 115.74, 78.97, 78.85, 59.51, 56.15, 50.92, 33.24; IR (ATR, cm^−1^): 3390 C(O)O-H (st), 3250 N-H (st), 3215 C-H alkyne (st), 2124 C≡C (st), 1888 armatic (st); MS (ESI^+^) *m*/*z*: [M]^+^ calculated for C_32_H_38_N_4_O_8_ 606.68, found 606.9.

#### 3.2.5. Synthesis of Tetrasubstituted EDTA G0-Alkyne (**8**)

The synthesis of compound **8** was performed according to a previously reported method with some modifications [43]. A mixture of compound **7** (1.18 g, 1.95 mmol), EDC·HCl (1.05 g, 5.46 mmol) and HOBt (0.60 g, 3.9 mmol) in DMF (6 mL) was stirred for 2 h at room temperature protected from light. Then, a solution of **4** (0.751 g, 4.29 mmol) in DMF (2 mL) was added dropwise. The resulting mixture was kept in the dark while stirring for 24 h at room temperature. After evaporation of the solvent under vacuum, the resulting solid was recrystallized from methanol, affording EDTA G0-alkyne **8** as a white powder (1.76 g, 1.92 mmol, yield 98%). ^1^H NMR (400 MHz, DMSO-*d*_6_, δ ppm): 8.18 (t, 4H, -CONH-), 7.12 (d, *J* = 8.22 Hz, 8H, He), 6.89 (d, *J* = 8.21 Hz, 8H, Hf), 4.74 (d, 8H, Hg), 3.54 (t, 4H, Hh), 3.30 (m, 8H, Hc), 3.06 (s, 8H, Hb), 2.68 (m, 8H, Hd), 2.53 (m, 4H, Ha); ^13^C-DEPTQ NMR (101 MHz, DMSO-*d*_6_, δ ppm): 170.92, 156.43, 132.82, 130.30, 115.48, 80.21, 78.88, 59.11, 56.12, 53.60, 40.12, 35.22; IR (ATR, cm^−1^): 3300 N-H (st), 3280 C-H alkyne (st), 2930, 2879 CH_2_ (st), 2832 C-H (st), 2130 C≡C (st), 1883 aromatic (st); MS (ESI^+^) *m*/*z*: [M + Na]^+^ calculated for C_54_H_59_N_6_NaO_8_ 943.09, found 943.

#### 3.2.6. Synthesis of Dendritic EDTA di-βCD

A solution of H_2_Asc (0.042 g, 0.23 mmol) in a DMSO:H_2_O mixture (1:1) (1 mL) was added dropwise to a solution of disubstituted EDTA alkyne **7** (0.50 g, 0.82 mmol), **6** (2.10 g, 1.81 mmol) and of CuSO_4_•5H_2_O (0.03 g, 0.12 mmol) in DMSO (8 mL), which was previously degassed by bubbling with nitrogen. The reaction mixture was heated to 80 °C with vigorous stirring under a nitrogen atmosphere for 12 h. At the end of this period, the reaction mixture was cooled and precipitated dropwise into cold acetone (200 mL). The formed solid formed was filtered under a vacuum. The obtained solid was purified by size exclusion chromatography through Bio-Gel^®^ P-6 medium using water as an eluent. The adequate fraction was lyophilized to obtain **EDTA di-βCD** as a beige solid (2.44 g, 0.83 mmol, 99%). *Conditions using microwave-assisted synthesis:* the microwave irradiation was performed using the same proportions as for the thermal methodology, under the following conditions: EDTA alkyne **7** (0.25 g, 0.41 mmol), **6** (1.05 g, 0.905 mmol), CuSO_4_•5H_2_O (0.015 g, 0.06 mmol), H_2_Asc (0.021 g, 0.115 mmol), DMSO:H_2_O (5.5:0.5) 6 mL, power 60 W, 30 min at 90 °C. This procedure permitted the rapid obtainment of dendritic **EDTA di-βCD** with the same yield. ^1^H NMR (400 MHz, DMSO-*d*_6_, δ ppm): 8.17 (br, 2H, H-triazole), 7.22 (br, 4H, He), 7.0 (m, 4H, Hf), 5.79 (br, 26H, OH-2,2′, OH-3,3′), 5.09 (s, 4H, Hg), 5.03 (s, 2H, H-1′), 4.87–4.77 (m, 18H, H-6, H-1, H-6′), 4.62 (br, 4H, OH-6, OH-6″), 3.99 (m, 2H, H-5), 3.64–3.56 (m, 60H, H-6, H-3,3′, H-5), 3.36–3.32 (m, 43H, Hc, H-4,4′, H-2,2′ overlapped with H_2_O), 3.08 (m, 4H, H-6″), 2.85 (m, 8H, H-b,b’,d,a); ^13^C-DEPTQ NMR (101 MHz, DMSO-*d*_6_, δ ppm): 171.32, 162.38, 143.35, 132.97, 130.60, 130.56, 125.16, 115.67, 102.73, 101.86, 84.25, 82.86, 82.15, 82.08, 73.84, 73.27, 72.55, 70.99, 60.70, 51.89, 51.06, 50.03; IR (ATR, cm^−1^): 3282 O-H (st), 2926 CH_2_ (st), 1023 C-O (st); MS (ESI-TOF^+^) *m*/*z*: [M + 2K]^+^ calculated for C_116_H_174_K_2_N_10_O_76_ 3002.86, found 1500.

#### 3.2.7. Synthesis of EDTA G0-βCD Dendrimer

According to the procedure described above for dendritic **EDTA di-βCD**, EDTA G0-alkyne **8** (0.20 g, 0.22 mmol), **6** (1.22 g, 1.05 mmol), CuSO_4_•5H_2_O (0.03 g, 0.12 mmol) and H_2_Asc (0.063 g, 0.35 mmol) were used to obtain of **EDTA G0-βCD** as beige solid (1.19 g, 0.22 mmol, 98%). *Conditions using microwave-assisted synthesis:* the microwave irradiation was performed using the same proportions as for the thermal methodology, under the following conditions: EDTA alkyne **8** (0.1 g, 0.11 mmol), **6** (0.61 g, 0.525 mmol), CuSO_4_•5H_2_O (0.015 g, 0.06 mmol), H_2_Asc (0.0315 g, 0.175 mmol), DMSO:H_2_O (5.5:0.5) 6 mL, power 60 W, 30 min at 90 °C. This procedure permitted the rapid obtainment of compound **EDTA G0-βCD** with the same yield. ^1^H NMR (400 MHz, DMSO-*d*_6_, δ ppm): 8.21 (br, 4H, -CONH-), 8.15 (s, 4H, H-triazole), 7.14 (d, *J* = 8.22 Hz, 8H, He), 6.96 (d, *J* = 8.21 Hz, 8H, Hf), 5.91–5.67 (m, 62H, OH-2,2′, OH-3,3′), 5.06 (br, 14H, Hg, H-1′), 4.92–4.79 (m, 31H, H-6′, H-1), 4.61–4.32 (m, 31H, OH-6, H-6′, OH-6″), 3.99 (m, 6H, H-5′), 3.73–3.59 (m, 97H, H-6, H-3,3′, H-5), 3.46–3.22 (m, 63H, H-4,4′, H-2,2′ overlapped with H_2_O), 3.14–3.10 (m, 13H, H-6″, Hc), 2.91 (m, 4H, H-6″), 2.68 (br, 10H, H-b,d), 2.57 (m, 15H, Ha overlapped with DMSO); ^13^C-DEPTQ NMR (101 MHz, DMSO-*d*_6_, δ ppm): 171.02, 157.40, 143.38, 132.33, 130.35, 126.36, 115.34, 102.72, 102.10, 84.21, 84.19, 82.84, 82.30, 73.80, 73.16, 72.55, 60.76, 54.66, 40.70, 33.42; IR (ATR, cm^−1^): 3306 O-H (st), 2928 CH_2_ (st), 1023 C-O (st); MS (MALDI-TOF^+^) *m*/*z*: [M + K]^+^ calculated for C_222_H_334_KN_18_NaO_144_ 5630, found 5629.67.

### 3.3. Determination of Water Solubility for EDTA di-βCD Dendritic and EDTA G0-βCD Dendrimer

The determination of the water solubility of the **EDTA-βCD**-based molecules was carried out according to a modified method reported by Jozwiakowski and Connors [59]. An adequate quantity of the compound was placed in three amber vials of 5 mL, with a magnetic stirring bar and a screw cap. The vials were filled with 1 mL of deionized water and sealed with parafilm to avoid water evaporation. The mixtures were stirred in an oil bath at a constant temperature of 25 ± 0.01 °C for 48 h. The supernatant was separated from the solid phase and filtered using a Milli-Q membrane (0.45 µm pore size) upon injection of the mixture from 3 mL volume disposable plastic syringes at 25 °C. The supernatant of each sample was lyophilized for 48 h, and the obtained solids were weighed on an analytical balance with an accuracy of ± 0.0001 g.

## 4. Conclusions

Herein, two PAMAM dendrimer analogs, a dendritic (**EDTA di-βCD)** and a G0 dendrimer (**EDTA G0-βCD)**, where the EDA core had been replaced by EDTA and decorated on their surfaces with two and four β-CD units, respectively, were reported. A synthetic method through click chemistry and microwave irradiation was used to obtain the final dendrimer compounds in high yields, with short reaction times and using simple purification procedures. This methodology is proposed as an efficient synthetic alternative for the obtention of a new class of dendrimers with EDTA core constructed through divergent synthesis, without Michael additions that permit the obtention of platforms with high solubility in water. According to their molecular characteristics, these dendrimers can be considered as potential drug delivery systems.

## Data Availability

Data are available within this article and in the associated Supplemental Materials.

## Supplementary Materials

The following supporting information can be downloaded at: https://www.mdpi.com/article/10.3390/pharmaceutics14112363/s1, Figure S1. ^1^H-NMR spectrum of *tert*-butyl (4-hydroxyphenethyl)carbamate. Figure S2. ^13^C-NMR spectrum of *tert*-butyl (4-hydroxyphenethyl)carbamate. Figure S3. IR spectrum of *tert*-butyl (4-hydroxyphenethyl)carbamate. Figure S4. DART *tert*-butyl (4-hydroxyphenethyl)carbamate. Figure S5. ^1^H-NMR spectrum of *tert*-butyl (4-(prop-2-yn-1-yloxy)phenyl)carbamate. Figure S6. ^13^C-NMR spectrum of *tert*-butyl (4-(prop-2-yn-1-yloxy)phenyl)carbamate. Figure S7. IR spectrum of *tert*-butyl (4-(prop-2-yn-1-yloxy)phenyl)carbamate. Figure S8. DART spectrum of *tert*-butyl (4-(prop-2-yn-1-yloxy)phenyl)carbamate. Figure S9. ^1^H-NMR spectrum of 2-(4-(prop-2-yn-1-yloxy)phenyl)ethan-1-amine. Figure S10. ^13^C-NMR spectrum of 2-(4-(prop-2-yn-1-yloxy)phenyl)ethan-1-amine. Figure S11. IR spectrum of 2-(4-(prop-2-yn-1-yloxy)phenyl)ethan-1-amine. Figure S12. DART spectrum of 2-(4-(prop-2-yn-1-yloxy)phenyl)ethan-1-amine. Figure S13. ^1^H-NMR spectrum of disubstituted EDTA alkyne. Figure S14. ^13^C-NMR spectrum of disubstituted EDTA alkyne. Figure S15. IR spectrum of disubstituted EDTA alkyne. Figure S16. ESI spectrum of disubstituted EDTA alkyne. Figure S17. ^1^H-NMR spectrum of tetrasubstituted EDTA G0-alkyne. Figure S18. ^13^C-NMR spectrum of tetrasubstituted EDTA G0-alkyne. Figure S19. IR spectrum of tetrasubstituted EDTA G0-alkyne. Figure S20. ESI spectrum of tetrasubstituted EDTA G0-alkyne. Figure S21. ^1^H-NMR spectrum of dendritic EDTA di-βCD. Figure S22. ^13^C-NMR spectrum of dendritic EDTA di-βCD. Figure S23. 2D NMR HMQC spectrum of dendritic EDTA di-βCD. Figure S24. 2D NMR COSY spectrum of dendritic EDTA di-βCD. Figure S25. IR spectrum of dendritic EDTA di-βCD. Figure S26. ESI-TOF spectrum of dendritic EDTA di-βCD. Figure S27. ^1^H-NMR spectrum of EDTA G0-βCD dendrimer. Figure S28. ^13^C-NMR spectrum of EDTA G0-βCD dendrimer. Figure S29. 2D NMR COSY spectrum of EDTA G0-βCD dendrimer. Figure S30. IR spectrum of EDTA G0-βCD dendrimer. Figure S31. MALDI-TOF spectrum of EDTA G0-βCD dendrimer.

## References

[1] Abbasi E., Aval S.F., Akbarzadeh A., Milani M., Nasrabadi H.T., Joo S.W., Hanifehpour Y., Nejati-Koshki K., Pashaei-Asl R. (2014). Dendrimers: Synthesis, Applications, and Properties. Nanoscale Res. Lett..

[2] Wu L.-P., Ficker M., Christensen J.B., Trohopoulos P.N., Moghimi S.M. (2015). Dendrimers in Medicine: Therapeutic Concepts and Pharmaceutical Challenges. Bioconjug. Chem..

[3] Maiti P.K., Çaǧın T., Wang G., Goddard W.A. (2004). Structure of PAMAM Dendrimers:  Generations 1 Through 11. Macromolecules.

[4] Mittal P., Saharan A., Verma R., Altalbawy F.M.A., Alfaidi M.A., Batiha G.E.-S., Akter W., Gautam K.R., Uddin S., Rahman S. (2021). Dendrimers: A New Race of Pharmaceutical Nanocarriers. BioMed Res. Int..

[5] Wang J., Lei L., Voets I.K., Stuart M.A., Velders A.H. (2020). Dendrimicelles with pH-Controlled Aggregation Number of Core-Dendrimers and Stability. Soft Matter.

[6] Matveev V.V., Markelov D.A., Dvinskikh S.V., Shishkin A.N., Tyutyukin K.V., Penkova A.V., Tatarinova E.A., Ignat’eva G.M., Milenin S.A. (2017). Investigation of Melts of Polybutylcarbosilane Dendrimers by ^1^H NMR Spectroscopy. Sci. Rep..

[7] Leiro V., Garcia J.P., Tomás H., Pêgo A.P. (2015). The Present and the Future of Degradable Dendrimers and Derivatives in Theranostics. Bioconjug. Chem..

[8] Sadekar S., Ghandehari H. (2012). Transepithelial Transport and Toxicity of PAMAM Dendrimers: Implications for Oral Drug Delivery. Adv. Drug Delivery Rev..

[9] Dong Z., Katsumi H., Sakane T., Yamamoto A. (2010). Effects of Polyamidoamine (PAMAM) Dendrimers on the Nasal Absorption of Poorly Absorbable Drugs in Rats. Int. J. Pharm..

[10] Cui T., Li S., Chen S., Liang Y., Sun H., Wang L. (2021). “Stealth” Dendrimers with Encapsulation of Indocyanine Green for Photothermal and Photodynamic Therapy of Cancer. Int. J. Pharm..

[11] Kim H., Choi B., Lim H., Min H., Oh J.H., Choi S., Cho J.G., Park J.-S., Lee S.J. (2017). Polyamidoamine Dendrimer-Conjugated Triamcinolone Acetonide Attenuates Nerve Injury-Induced Spinal Cord Microglia Activation and Mechanical Allodynia. Mol. Pain.

[12] Gorzkiewicz M., Janaszewska A., Ficker M., Svenningsen S.W., Christensen J.B., Klajnert-Maculewicz B. (2019). Pyrrolidone-modified PAMAM Dendrimers Enhance Anti-Inflammatory Potential of Indomethacin in vitro. Colloids Surf. B.

[13] Mendes P.L., Pan J., Torchilin V.P. (2017). Dendrimers as Nanocarriers for Nucleic Acid and Drug Delivery in Cancer Therapy. Molecules.

[14] Noriega-Luna B., Godínez L.A., Rodríguez F.J., Rodríguez A., Zaldívar-Lelo de Larrea G., Sosa-Ferreyra C.F., Mercado-Curiel R.F., Manríquez J., Bustos E. (2014). Applications of Dendrimers in Drug Delivery Agents, Diagnosis, Therapy, and Detection. J. Nanomater..

[15] Barrett T., Ravizzini G., Choyke P.L., Kobayashi H. (2009). Dendrimers in Medical Nanotechnology. IEEE Eng. Med. Biol. Mag..

[16] Gorzkiewicz M., Kopeć O., Janaszewska A., Konopka M., Pędziwiatr-Werbicka E., Tarasenko I.I., Bezrodnyi V.V., Neelov I.M., Klajnert-Maculewicz B. (2020). Poly(Lysine) Dendrimers Form Complexes with siRNA and Provide its Efficient Uptake by Myeloid Cells: Model Studies for Therapeutic Nucleic Acid Delivery. Int. J. Mol. Sci..

[17] Idris A.O., Mamba B., Feleni U. (2020). Poly (Propylene Imine) Dendrimer: A Potential Nanomaterial for Electrochemical Application. Mater. Chem. Phys..

[18] Singh V., Sahebkar A., Kesharwani P. (2021). Poly (Propylene Imine) Dendrimer as an Emerging Polymeric Nanocarrier for Anticancer Drug and Gene Delivery. Eur. Polym. J..

[19] Cangiotti M., Staneva D., Ottaviani M.F., Vasileva-Tonkova E., Grabchev I. (2021). Synthesis and Characterization of Fluorescent PAMAM Dendrimer Modified with 1,8-Naphthalimide Units and its Cu(II) Complex Designed for Specific Biomedical Application. J. Photochem. Photobiol. A.

[20] Jose J., Charyulu R.N. (2016). Prolonged Drug Delivery System of an Antifungal Drug by Association with Polyamidoamine Dendrimers. Int. J. Pharm. Investig..

[21] Villamagna I.J., Gordon T.N., Hurtig M.B., Beier F., Gillies E.R. (2019). Poly(Ester Amide) Particles for Controlled Delivery of Celecoxib. J. Biomed. Mater. Res. Part A.

[22] Lancelot A., González-Pastor R., Clavería-Gimeno R., Romero P., Abian O., Martín-Duque P., Serrano J.L., Sierra T. (2018). Cationic Poly(Ester Amide) Dendrimers: Alluring Materials for Biomedical Applications. J. Mater. Chem. B.

[23] Caminade A.-M., Maraval V., Laurent R., Turrin C.-O., Sutra P., Leclaire J., Griffe L., Marchand P., Baudoin-Dehoux C., Rebout C. (2003). Phosphorus Dendrimers: From Synthesis to Applications. Comptes Rendus Chim..

[24] Mignani S., Shi X., Ceña V., Shcharbin D., Bryszewska M., Majoral J.-P. (2021). In vivo Therapeutic Applications of Phosphorus Dendrimers: State of the Art. Drug Discov. Today.

[25] Lyu Z., Ding L., Huang A.Y.-T., Kao C.-L., Peng L. (2019). Poly(Amidoamine) Dendrimers: Covalent and Supramolecular Synthesis. Mater. Today Chem..

[26] Kaur D., Jain K., Mehra N.K., Kesharwani P., Jain N.K. (2016). A Review on Comparative Study of PPI and PAMAM Dendrimers. J. Nanopart. Res..

[27] Boas U., Heegaard P.M.H. (2004). Dendrimers in Drug Research. Chem. Soc. Rev..

[28] Sebestik J., Niederhafner P., Jezek J. (2011). Peptide and Glycopeptide Dendrimers and Analogous Dendrimeric Structures and their Biomedical Applications. Amino Acids.

[29] de Araújo R.V., da Silva Santos S., Igne Ferreira E., Giarolla J. (2018). New Advances in General Biomedical Applications of PAMAM Dendrimers. Molecules.

[30] Dias A.P., da Silva Santos S., da Silva J.V., Parise-Filho R., Igne Ferreira E., El Seoud O., Giarolla J. (2020). Dendrimers in the Context of Nanomedicine. Int. J. Pharm..

[31] Nwe K., Milenic D.E., Ray G.L., Kim Y.-S., Brechbiel M.W. (2012). Preparation of Cystamine Core Dendrimer and Antibody–Dendrimer Conjugates for MRI Angiography. Mol. Pharm..

[32] Gupta V., Nayak S.K. (2015). Dendrimers: A Review on Synthetic Approaches. J. Appl. Pharm. Sci..

[33] Kharwade R., More S., Warokar A., Agrawal P., Mahajan N. (2020). Starburst Pamam Dendrimers: Synthetic Approaches, Surface Modifications, and Biomedical Applications. Arab. J. Chem..

[34] Peterson J., Allikmaa V., Subbi J., Pehk T., Lopp M. (2003). Structural Deviations in Poly(Amidoamine) Dendrimers: A MALDI-TOF MS Analysis. Eur. Polym. J..

[35] Sánchez-Navarro M., Rojo J., de la Fuente J.M., Grazu V. (2012). Chapter 5—Synthetic Strategies to Create Dendrimers: Advantages and Drawbacks. Nanobiotechnology: Inorganic Nanoparticles vs Organic Nanoparticles.

[36] Shaunak S. (2015). Perspective: Dendrimer Drugs for Infection and Inflammation. Biochem. Biophys. Res. Commun..

[37] Thomas T.P., Huang B., Choi S.K., Silpe J.E., Kotlyar A., Desai A.M., Zong H., Gam J., Joice M., Baker J.R. (2012). Polyvalent Dendrimer-Methotrexate as a Folate Receptor-Targeted Cancer Therapeutic. Mol. Pharm..

[38] Fox L.J., Richardson R.M., Briscoe W.H. (2018). PAMAM Dendrimer-Cell Membrane Interactions. Adv. Colloid Interface Sci..

[39] Janaszewska A., Lazniewska J., Trzepiński P., Marcinkowska M., Klajnert-Maculewicz B. (2019). Cytotoxicity of Dendrimers. Biomolecules.

[40] Chis A.A., Dobrea C., Morgovan C., Arseniu A.M., Rus L.L., Butuca A., Juncan A.M., Totan M., Vonica-Tincu A.L., Cormos G. (2020). Applications and Limitations of Dendrimers in Biomedicine. Molecules.

[41] Mishra V., Gupta U., Jain N.K. (2009). Surface-Engineered Dendrimers: A Solution for Toxicity Issues. J. Biomater. Sci. Polym. Ed..

[42] Yang J., Zhang Q., Chang H., Cheng Y. (2015). Surface-Engineered Dendrimers in Gene Delivery. Chem. Rev..

[43] González-Méndez I., Hameau A., Laurent R., Bijani C., Bourdon V., Caminade A.-M., Rivera E., Moineau-Chane Ching K.I. (2020). β-Cyclodextrin PAMAM Dendrimer: How to Overcome the Tumbling Process for Getting Fully Available Host Cavities. Eur. J. Org. Chem..

[44] Sorroza-Martínez K., González-Méndez I., Martínez-Serrano R.D., Solano J.D., Ruiu A., Illescas J., Zhu X.X., Rivera E. (2020). Efficient Modification of PAMAM G1 Dendrimer Surface with β-Cyclodextrin Units by CuAAC: Impact on the Water Solubility and Cytotoxicity. RSC Adv..

[45] Przybyla M.A., Yilmaz G., Becer C.R. (2020). Natural Cyclodextrins and their Derivatives for Polymer Synthesis. Polym. Chem..

[46] Arima H. (2021). Twenty Years of Research on Cyclodextrin Conjugates with PAMAM Dendrimers. Pharmaceutics.

[47] Qiu J., Kong L., Cao X., Li A., Tan H., Shi X. (2016). Dendrimer-Entrapped Gold Nanoparticles Modified with β-Cyclodextrin for Enhanced Gene Delivery Applications. RSC Adv..

[48] Menjoge A.R., Kannan R.M., Tomalia D.A. (2010). Dendrimer-based drug and imaging conjugates: Design considerations for nanomedical applications. Drug Discov. Today.

[49] Buschhaus B., Hampel F., Grimme S., Hirsch A. (2005). Metal-Induced Chiral folding of Depsipeptide Dendrimers. Chem. Eur. J..

[50] Ramírez-Palma M.T., Apolonio V.M., González J., Martínez-Barrera G., Corona D., Cuevas-Yañez E. (2017). Synthesis of EDTA Core Dendrimers Through a Consecutive Esterification-CuAAC Process. J. Macromol. Sci. Part A Pure Appl. Chem..

[51] Hameau A., Fuchs S., Laurent R., Majoral J.-P., Caminade A.-M. (2011). Synthesis of Dye/Fluorescent Functionalized Dendrons Based on Cyclotriphosphazene. Beilstein J. Org. Chem..

[52] Cheng M.H.Y., Savoie H., Bryden F., Boyle R.W. (2017). A Convenient Method for Multicolour Labelling of Proteins with BODIPY Fluorophores via Tyrosine Residues. Photochem. Photobiol. Sci..

[53] Ting C.-H., Chen J.-T., Hsu C.-S. (2002). Synthesis and Thermal and Photoluminescence Properties of Liquid Crystalline Polyacetylenes Containing 4-Alkanyloxyphenyl *trans*-4-Alkylcyclohexanoate Side Groups. Macromolecules.

[54] Li Z., Huang R., Xu H., Chen J., Zhan Y., Zhou X., Chen H., Jiang B. (2017). Divinylsulfonamides as Specific Linkers for Stapling Disulfide Bonds in Peptides. Org. Lett..

[55] Antelo A., Jover A., Galantini L., Meijide F., Alvarez Alcalde M., Viorel Pavel N., Vázquez Tato J. (2011). Formation of Host-Guest and Sandwich Complexes by a β-Cyclodextrin Derivative. J. Incl. Phenom. Macrocycl. Chem..

[56] Zhong N., Byun H.-S., Bittman R. (1998). An Improved Synthesis of 6-*O*-Monotosyl-6-Deoxy-β-Cyclodextrin. Tetrahedron Lett..

[57] Liu H., Zhang Y., Hu J., Li C., Liu S. (2009). Multi-Responsive Supramolecular Double Hydrophilic Diblock Copolymer Driven by Host-Guest Inclusion Complexation between *β*-Cyclodextrin and Adamantyl Moieties. Macromol. Chem. Phys..

[58] Sorroza-Martínez K., González-Méndez I., Vonlanthen M., Moineau-Chane Ching K.I., Caminade A.-M., Illescas J., Rivera E. (2020). First Class of Phosphorus Dendritic Compounds Containing β-Cyclodextrin Units in the Periphery Prepared by CuAAC. Molecules.

[59] Jozwiakowski M.J., Connors K.A. (1985). Aqueous solubility behavior of three cyclodextrins. Carbohydr. Res..

