# Supplementary material for: Synthesis of β-Cyclodextrin-Decorated Dendritic Compounds Based on EDTA Core: A New Class of PAMAM Dendrimer Analogs
Source: Pharmaceutics. 2022 Nov 2;14(11):2363. doi: 10.3390/pharmaceutics14112363 (PMC9697223; doi:10.3390/pharmaceutics14112363)
Supplement: Supplementary file 1 [file pharmaceutics-14-02363-s001.zip › pharmaceutics-1960748-supplementary.pdf]

Synthesis of β-cyclodextrin-decorated dendritic compounds based on EDTA core: a new class of PAMAM dendrimer analogs

Israel González-Méndez [1], Esteban Loera-Loera [1, 2], Kendra Sorroza-Martínez [3], Mireille Vonlanthen [3], Fabián Cuétara-Guadarrama [3], María Josefa Bernad-Bernad [4], Ernesto Rivera [3,*] and Jesús Gracia-Mora [1,].**

Supplementary Information

Analysis data of

Figure S1. ^{1}H-NMR spectrum of *tert*-butyl (4-hydroxyphenethyl)carbamate. p3

Figure S2. ^{13}C-NMR spectrum of *tert*-butyl (4-hydroxyphenethyl)carbamate. p3

Figure S3. IR spectrum of *tert*-butyl (4-hydroxyphenethyl)carbamate. p4

Figure S4. DART spectrum of *tert*-butyl (4-hydroxyphenethyl)carbamate. p4

Figure S5. ^{1}H-NMR spectrum of *tert*-butyl (4-(prop-2-yn-1-yloxy)phenyl)carbamate. p5

Figure S6. ^{13}C-NMR spectrum of *tert*-butyl (4-(prop-2-yn-1-yloxy)phenyl)carbamate. p5

Figure S7. IR spectrum of *tert*-butyl (4-(prop-2-yn-1-yloxy)phenyl)carbamate. p6

Figure S8. DART spectrum of *tert*-butyl (4-(prop-2-yn-1-yloxy)phenyl)carbamate. p6

Figure S9. ^{1}H-NMR spectrum of 2-(4-(prop-2-yn-1-yloxy)phenyl)ethan-1-amine. p7

Figure S10. ^{13}C-NMR spectrum of 2-(4-(prop-2-yn-1-yloxy)phenyl)ethan-1-amine. p7

Figure S11. IR spectrum of 2-(4-(prop-2-yn-1-yloxy)phenyl)ethan-1-amine. p8

Figure S12. DART spectrum of 2-(4-(prop-2-yn-1-yloxy)phenyl)ethan-1-amine. p8

Figure S13. ^{1}H-NMR spectrum of disubstituted EDTA alkyne. p9

Figure S14. ^{13}C-NMR spectrum of disubstituted EDTA alkyne. p9

Figure S15. IR spectrum of disubstituted EDTA alkyne. p10

Figure S16. ESI spectrum of disubstituted EDTA alkyne. p10

Figure S17. ^{1}H-NMR spectrum of tetrasubstituted EDTA G0-alkyne. p11

Figure S18. ^{13}C-NMR spectrum of tetrasubstituted EDTA G0-alkyne. p11

Figure S19. IR spectrum of tetrasubstituted EDTA G0-alkyne. p12

Figure S20. ESI spectrum of tetrasubstituted EDTA G0-alkyne. p12

Figure S21. ^{1}H-NMR spectrum of dendritic EDTA di-βCD. p13

Figure S22. ^{13}C-NMR spectrum of dendritic EDTA di-βCD. p13

Figure S23. 2D NMR HMQC spectrum of dendritic EDTA di-βCD. p14

Figure S24. 2D NMR COSY spectrum of dendritic EDTA di-βCD. p14

Figure S25. IR spectrum of dendritic EDTA di-βCD. p15

Figure S26. ESI-TOF spectrum of dendritic EDTA di-βCD. p15

Figure S27. ^{1}H-NMR spectrum of EDTA G0-βCD dendrimer. p16

Figure S28. ^{13}C-NMR spectrum of EDTA G0-βCD dendrimer. p16

Figure S29. 2D NMR COSY spectrum of EDTA G0-βCD dendrimer. p17

Figure S30. IR spectrum of EDTA G0-βCD dendrimer. p17

Figure S31. MALDI spectrum of EDTA G0-βCD dendrimer. p18

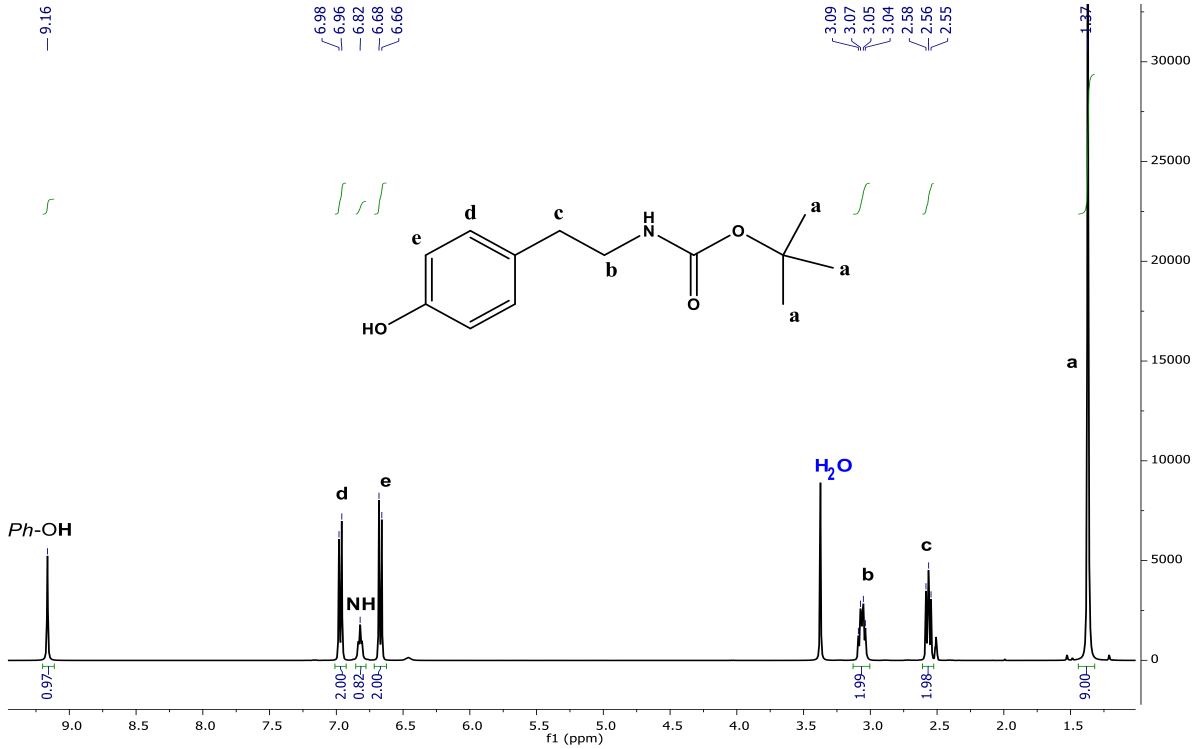

Figure S1. ^{1}H-NMR spectrum of *tert*-butyl (4-hydroxyphenethyl)carbamate.

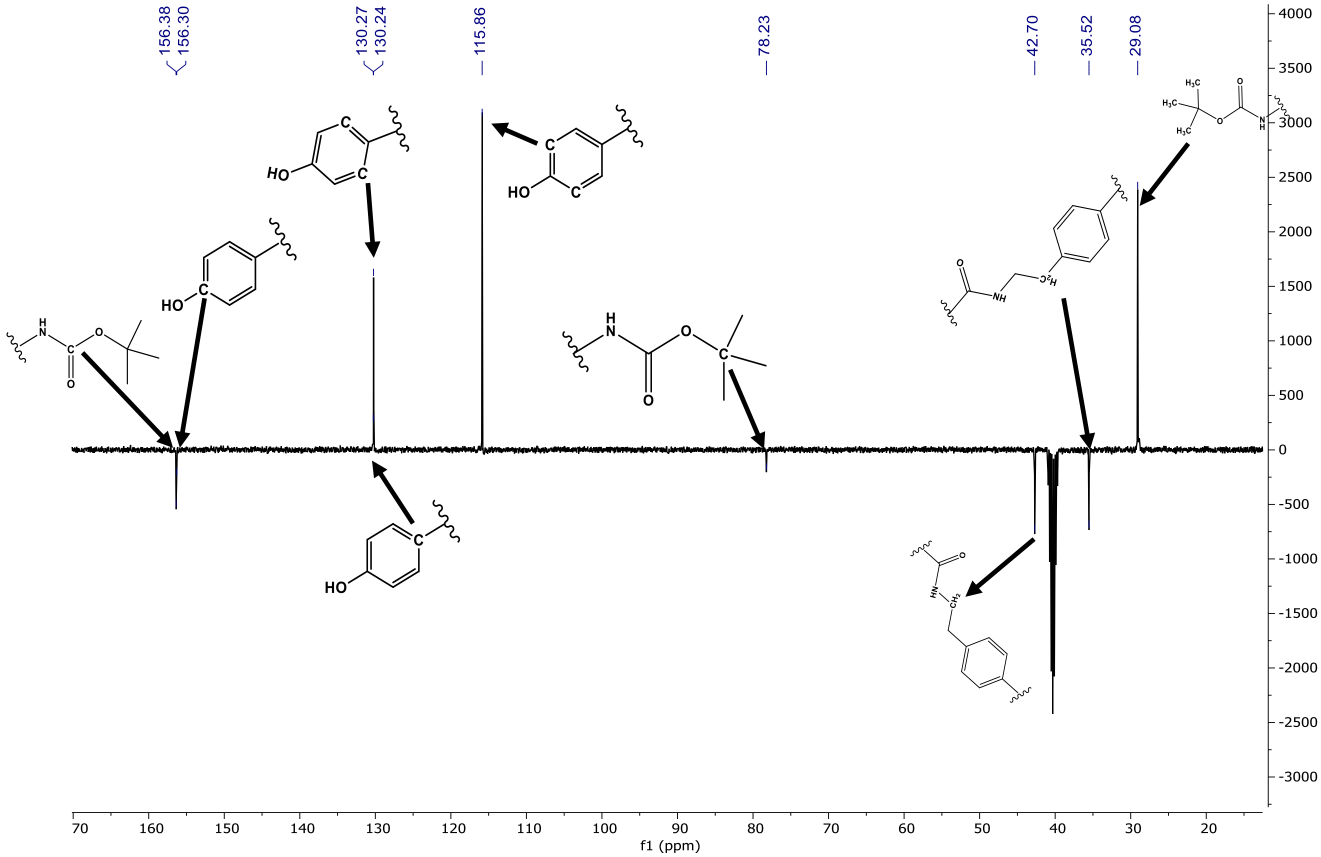

Figure S2. ^{13}C-NMR spectrum of *tert*-butyl (4-hydroxyphenethyl)carbamate.

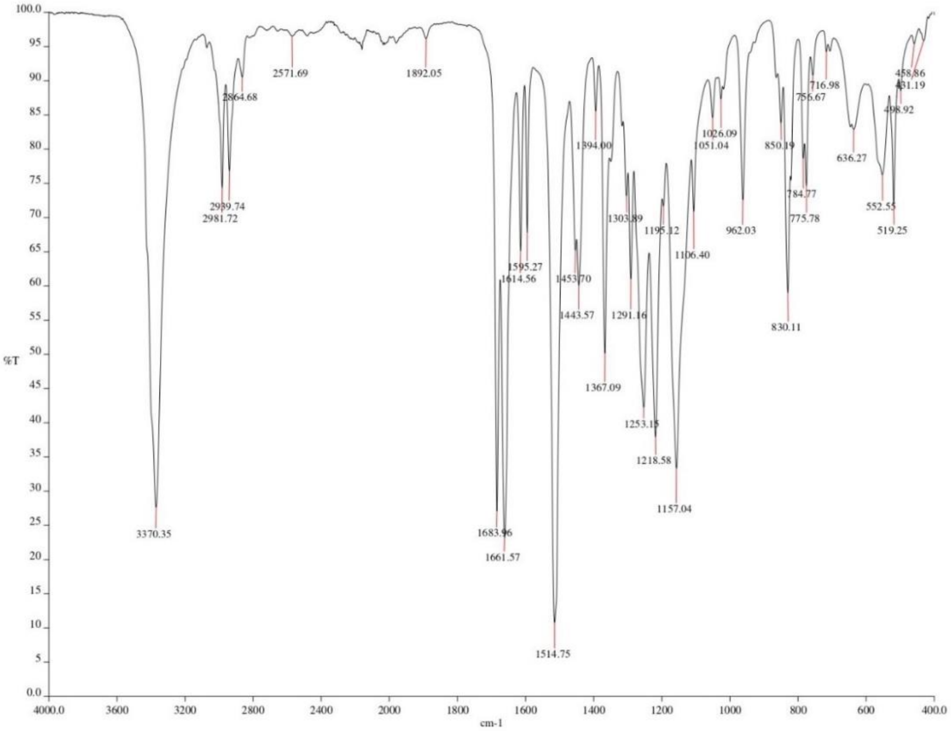

Figure S3. IR spectrum of *tert*-butyl (4-hydroxyphenethyl)carbamate.

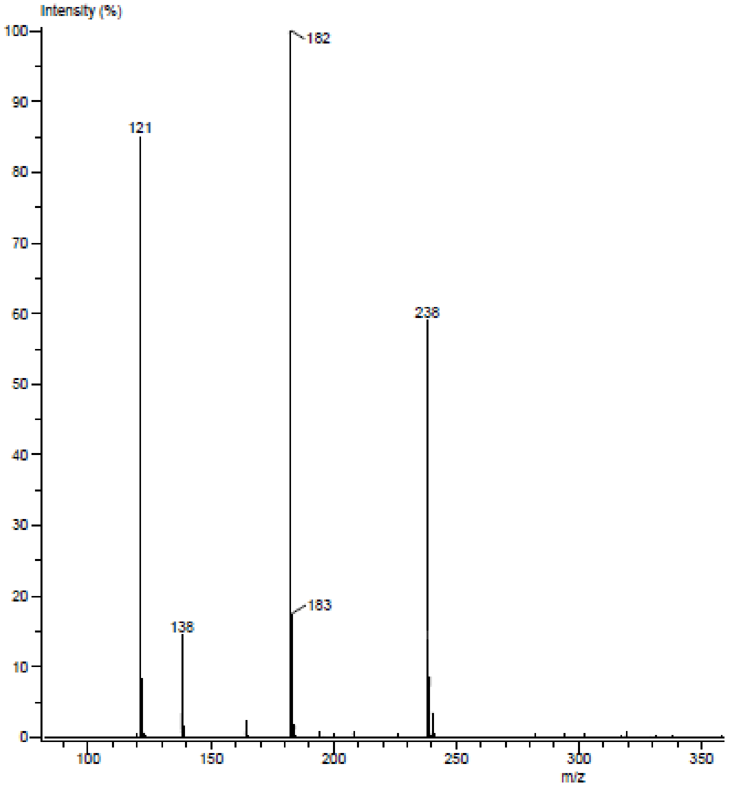

Figure S4. DART *tert*-butyl (4-hydroxyphenethyl)carbamate.

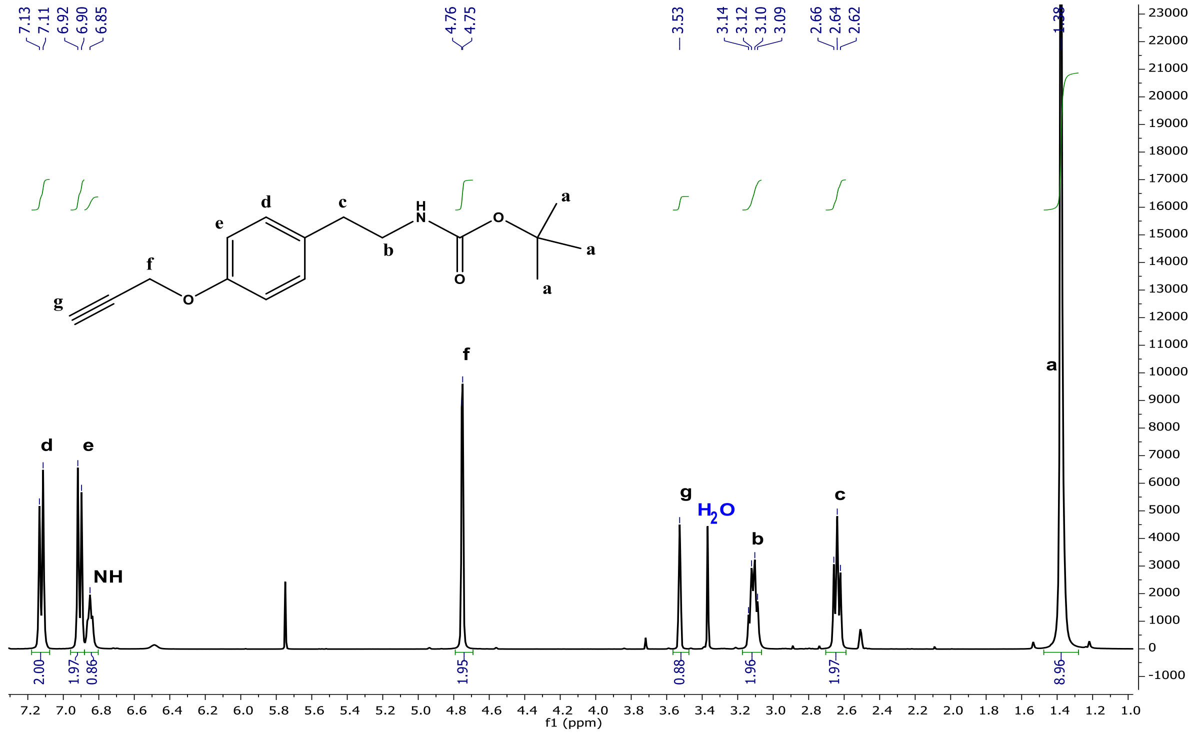

Figure S5. ^{1}H-NMR spectrum of *tert*-butyl (4-(prop-2-yn-1-yloxy)phenyl)carbamate.

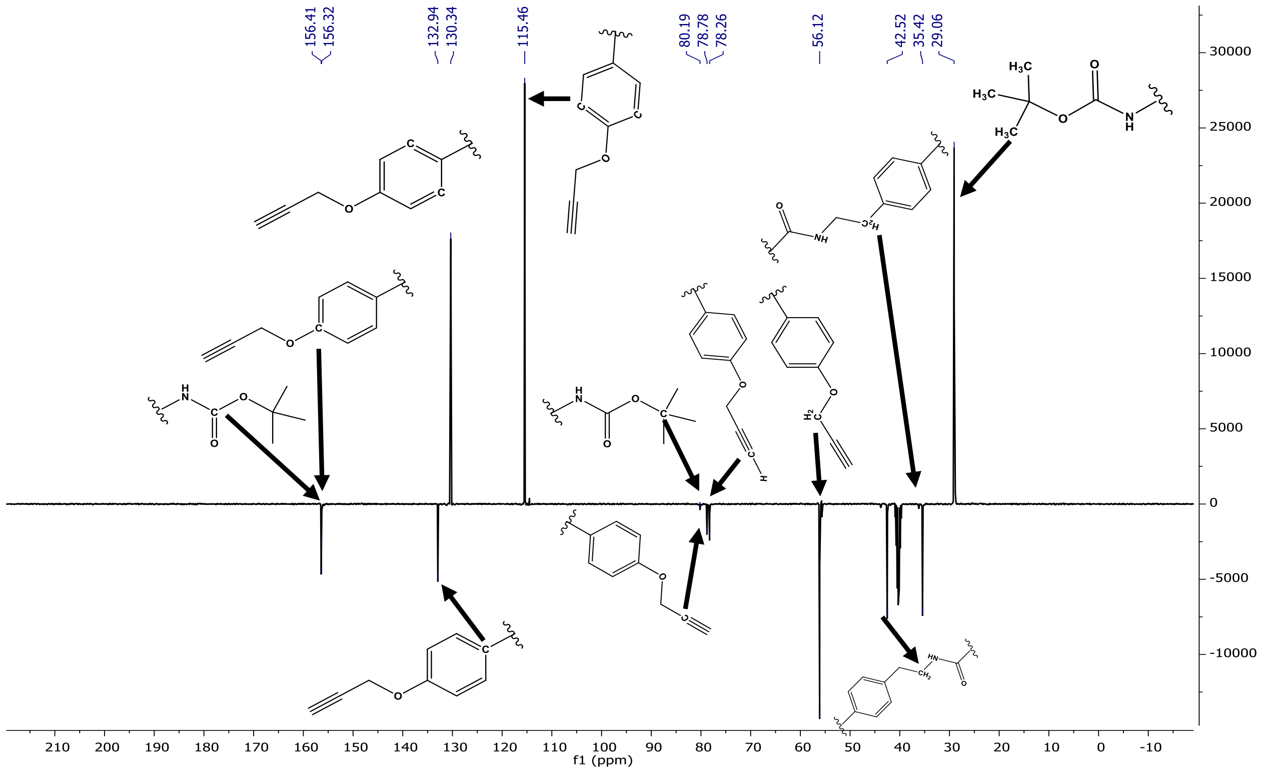

Figure S6. ^{13}C-NMR spectrum of *tert*-butyl (4-(prop-2-yn-1-yloxy)phenyl)carbamate.

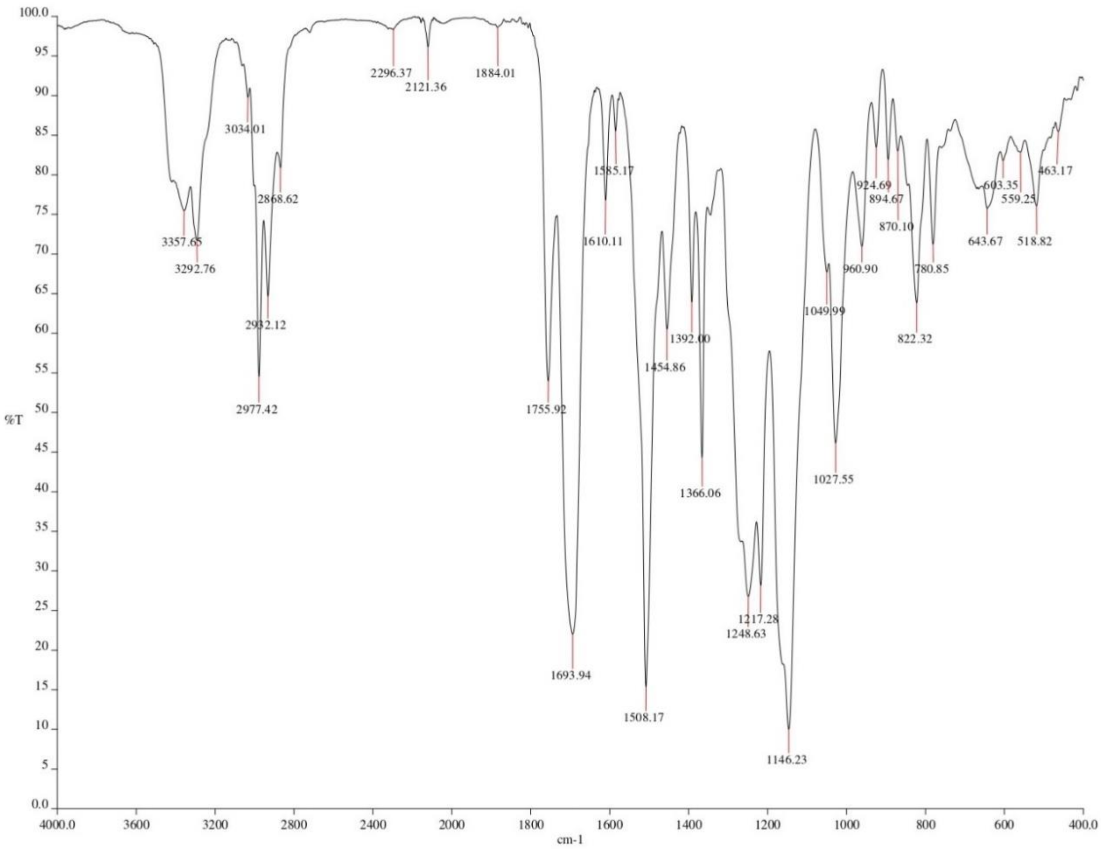

Figure S7. IR spectrum of *tert*-butyl (4-(prop-2-yn-1-yloxy)phenyl)carbamate.

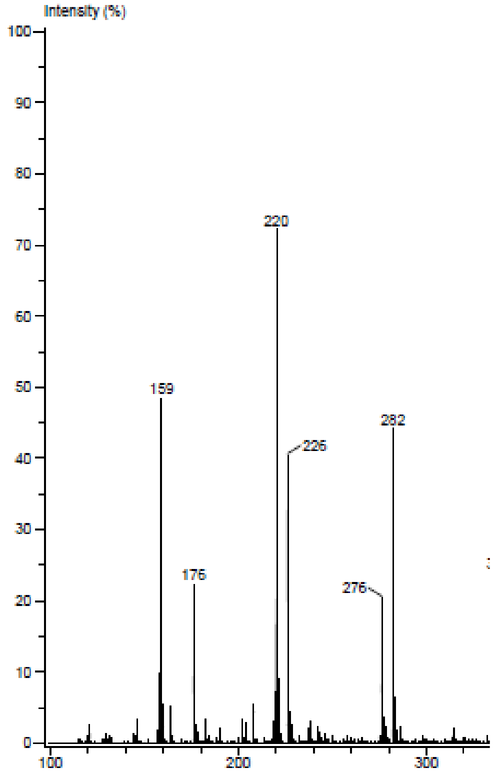

Figure S8. DART spectrum of *tert*-butyl (4-(prop-2-yn-1-yloxy)phenyl)carbamate.

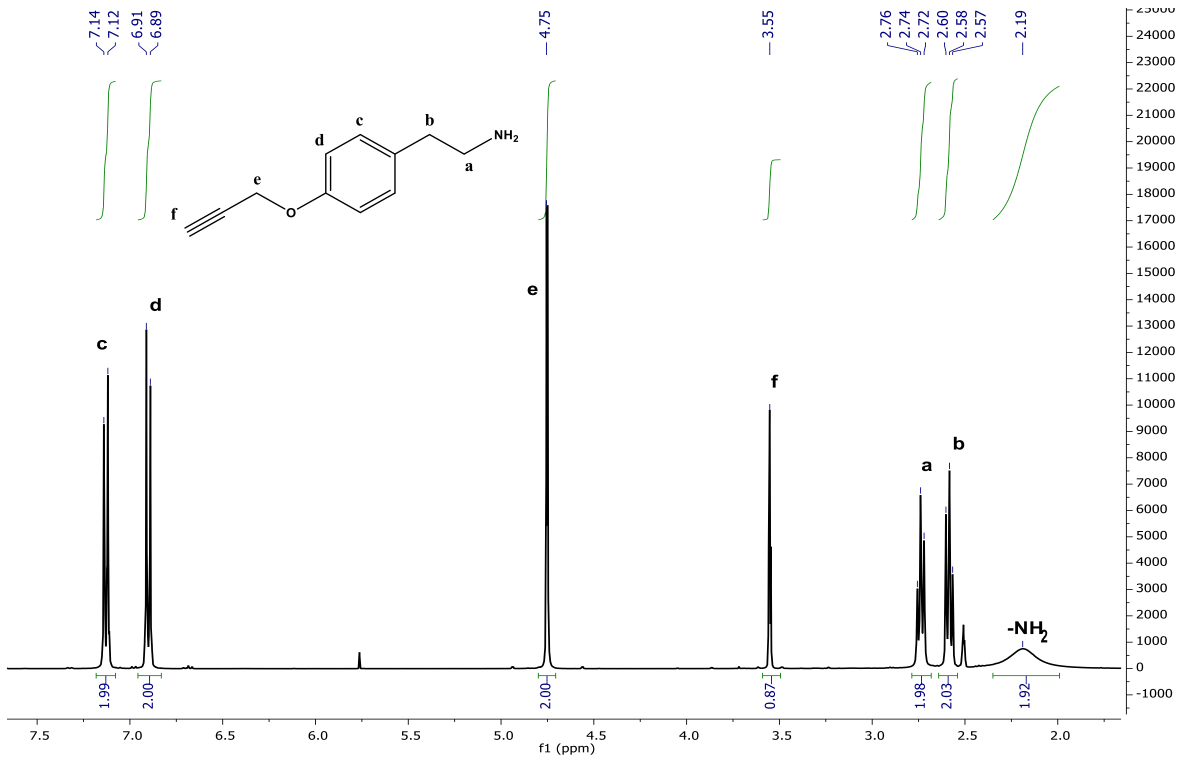

Figure S9. ^{1}H-NMR spectrum of 2-(4-(prop-2-yn-1-yloxy)phenyl)ethan-1-amine.

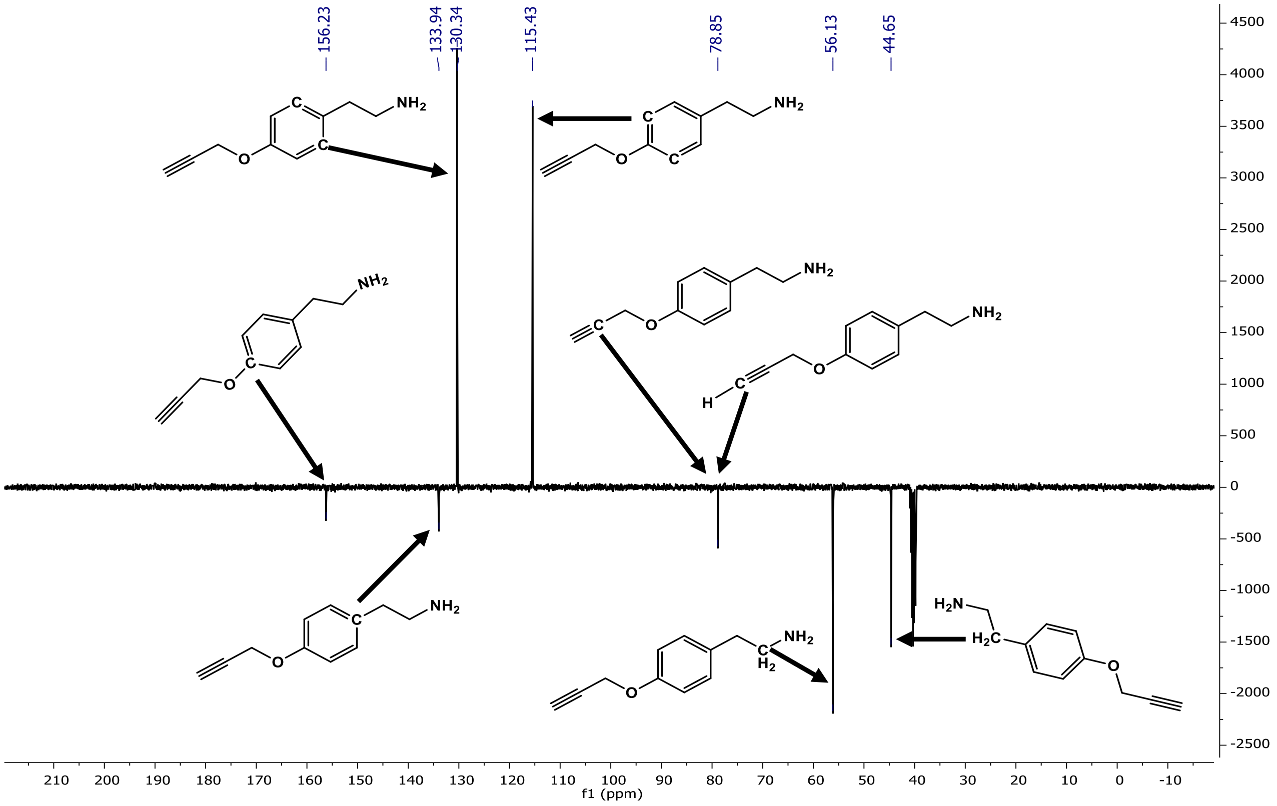

Figure S10. ^{13}C-NMR spectrum of 2-(4-(prop-2-yn-1-yloxy)phenyl)ethan-1-amine.

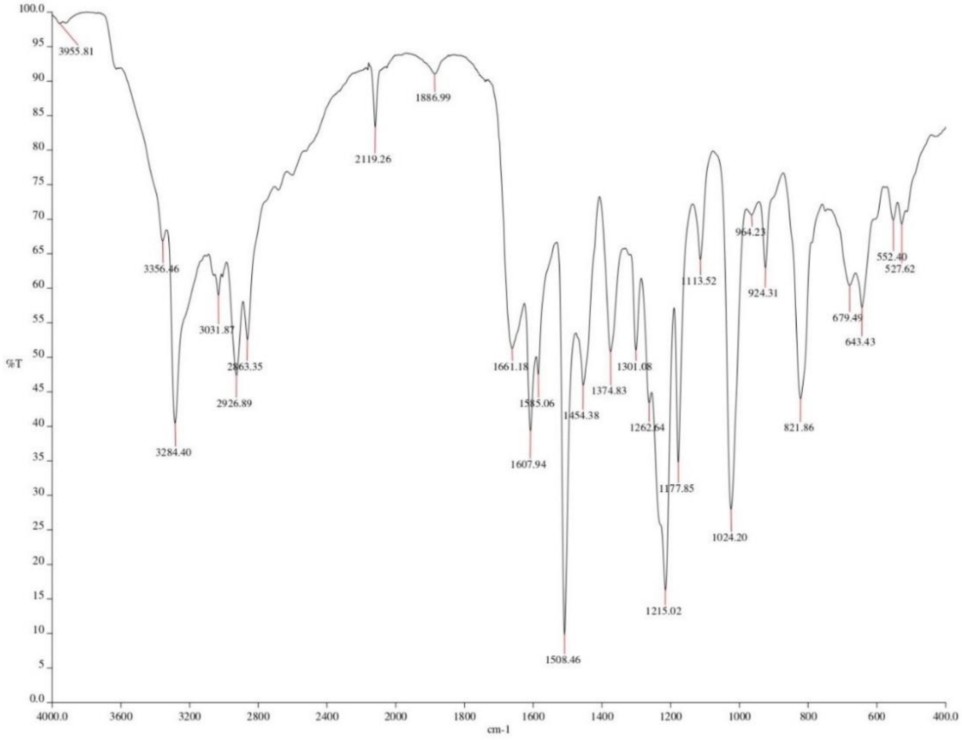

Figure S11. IR spectrum of 2-(4-(prop-2-yn-1-yloxy)phenyl)ethan-1-amine.

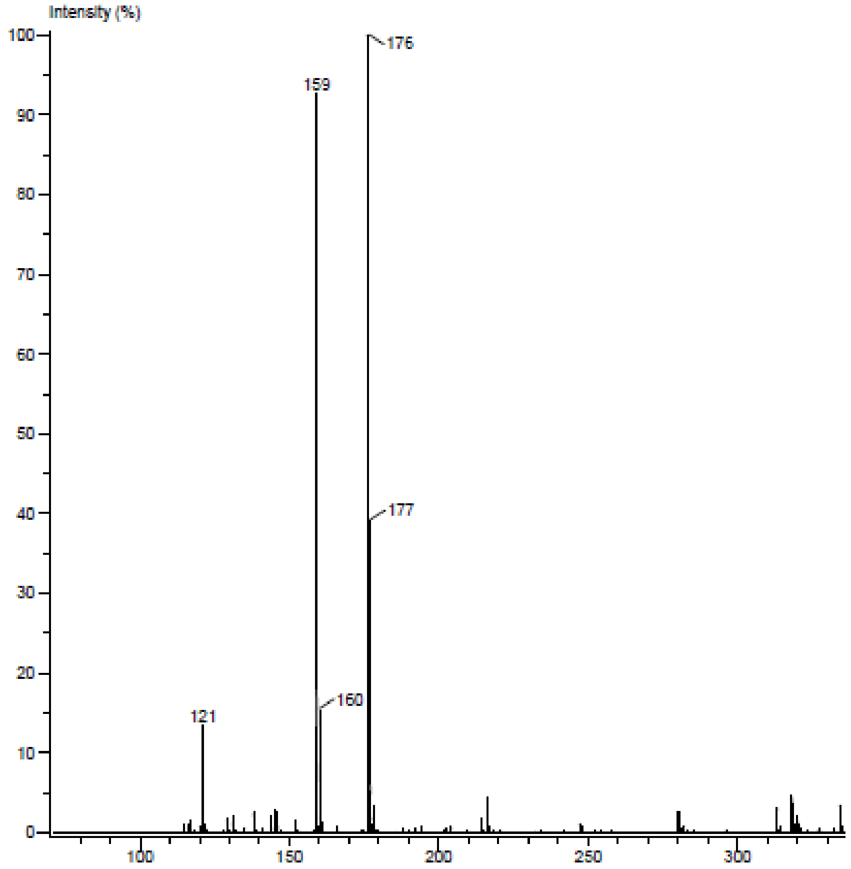

Figure S12. DART spectrum of 2-(4-(prop-2-yn-1-yloxy)phenyl)ethan-1-amine.

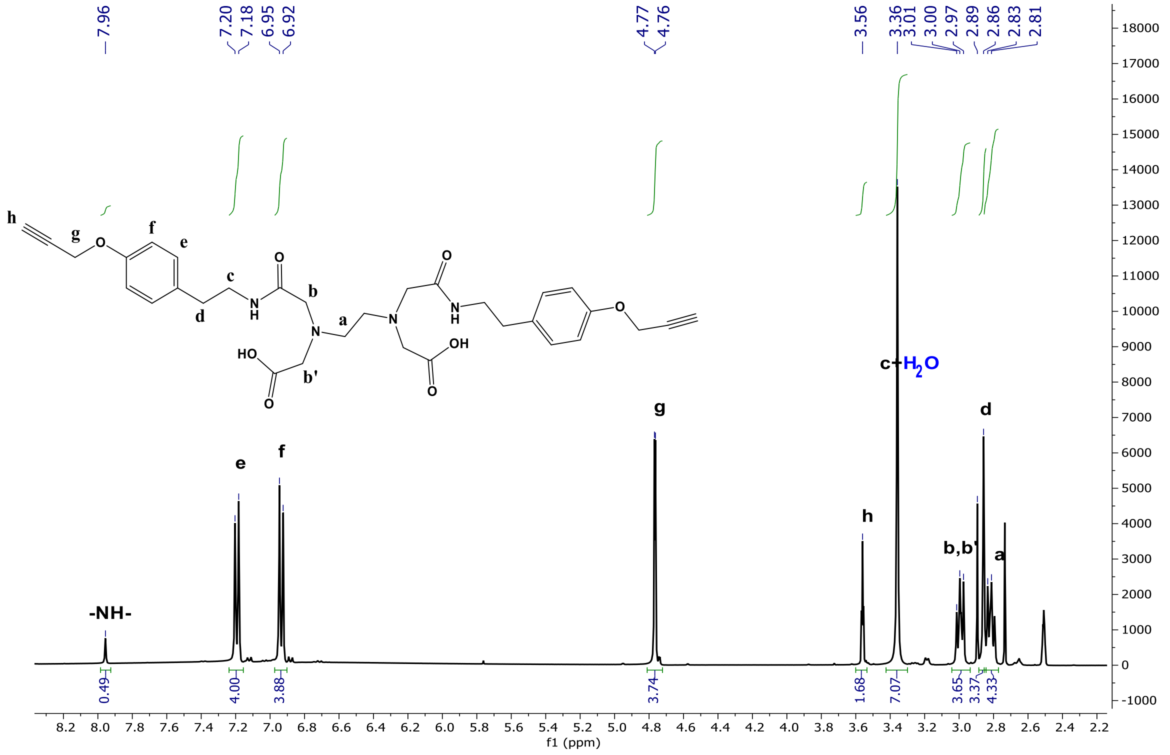

Figure S13. ^{1}H-NMR spectrum of disubstituted EDTA alkyne.

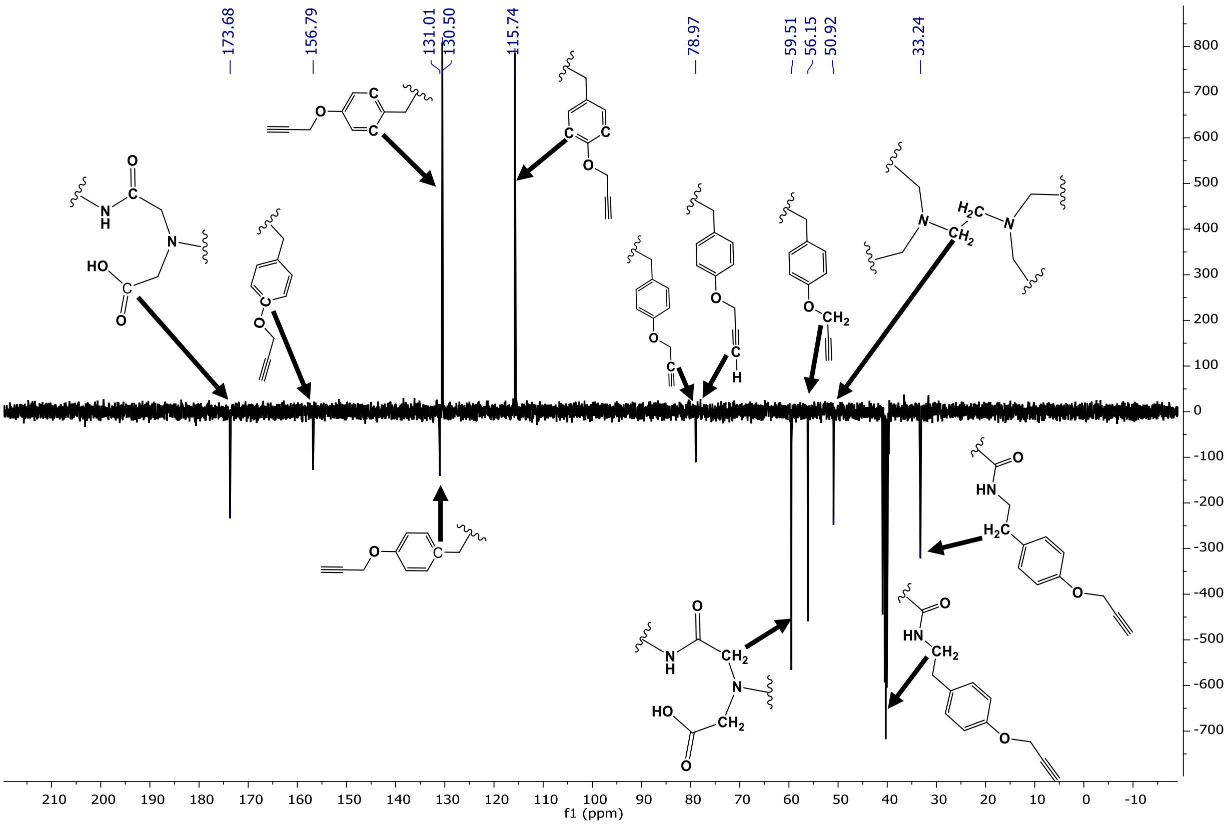

Figure S14. ^{13}C-NMR spectrum of disubstituted EDTA alkyne.

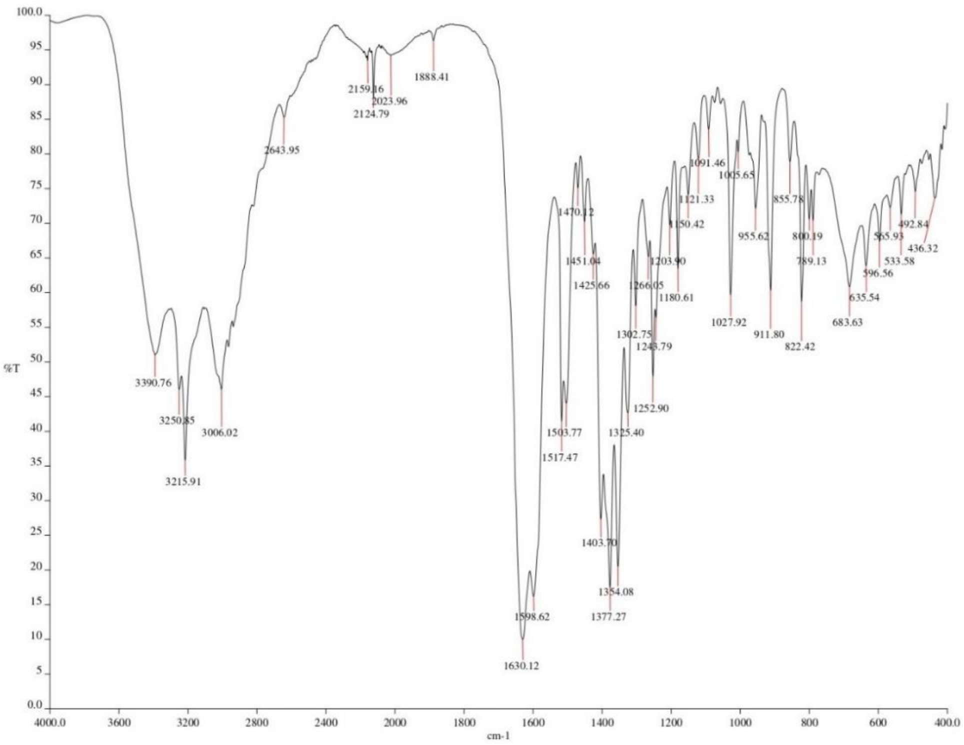

Figure S15. IR spectrum of disubstituted EDTA alkyne.

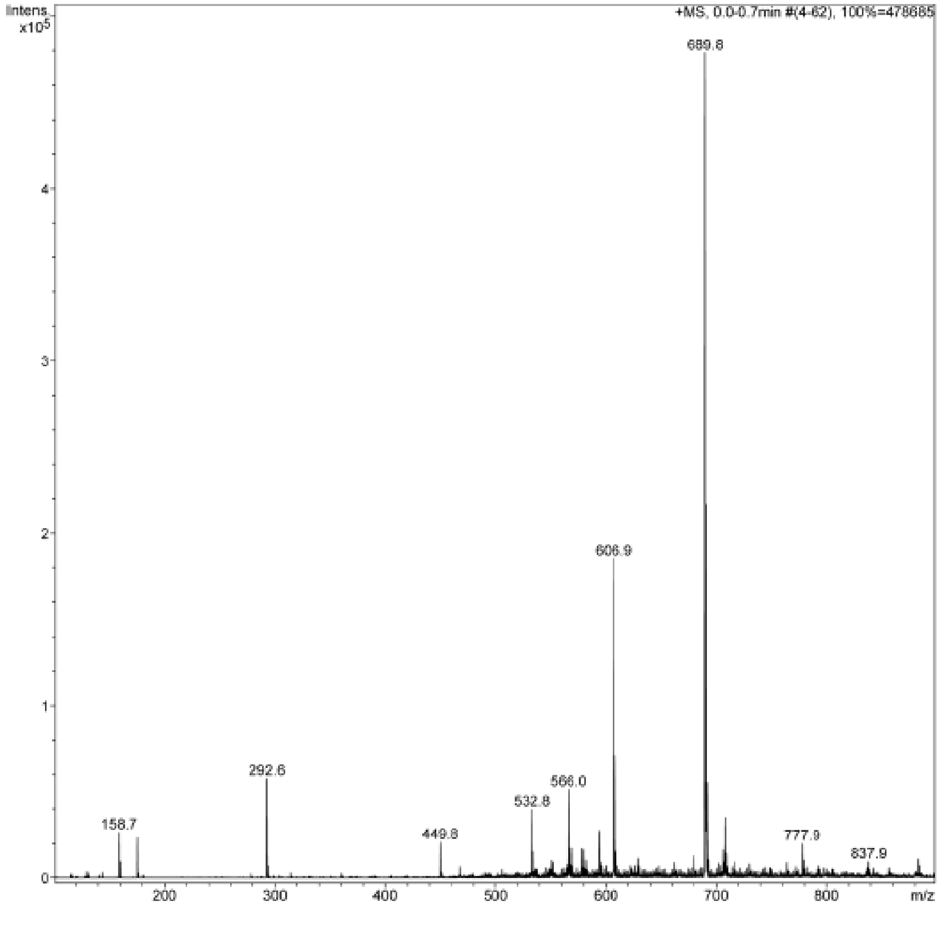

Figure S16. ESI spectrum of disubstituted EDTA alkyne.

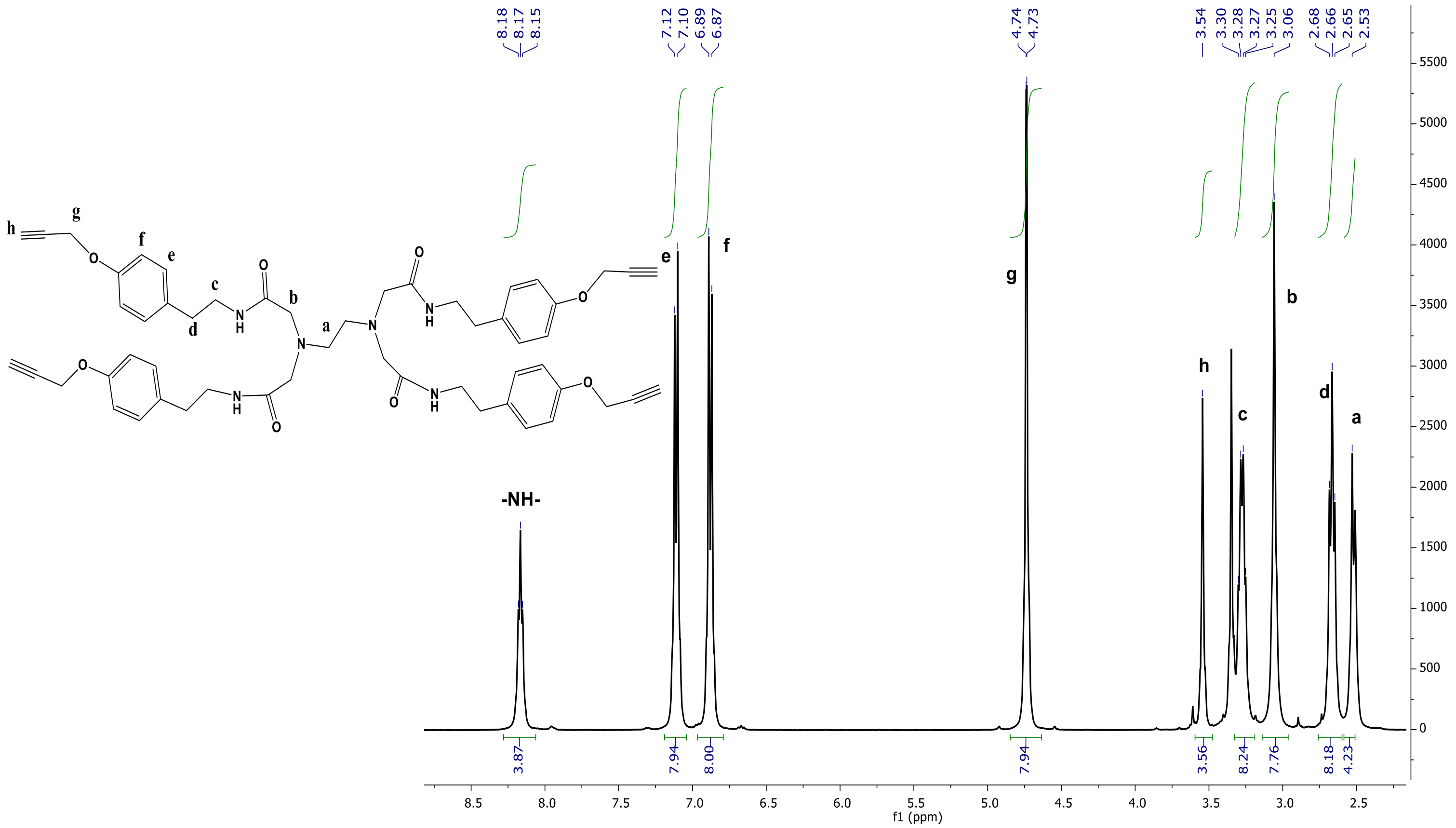

Figure S17. ^{1}H-NMR spectrum of tetrasubstituted EDTA G0-alkyne.

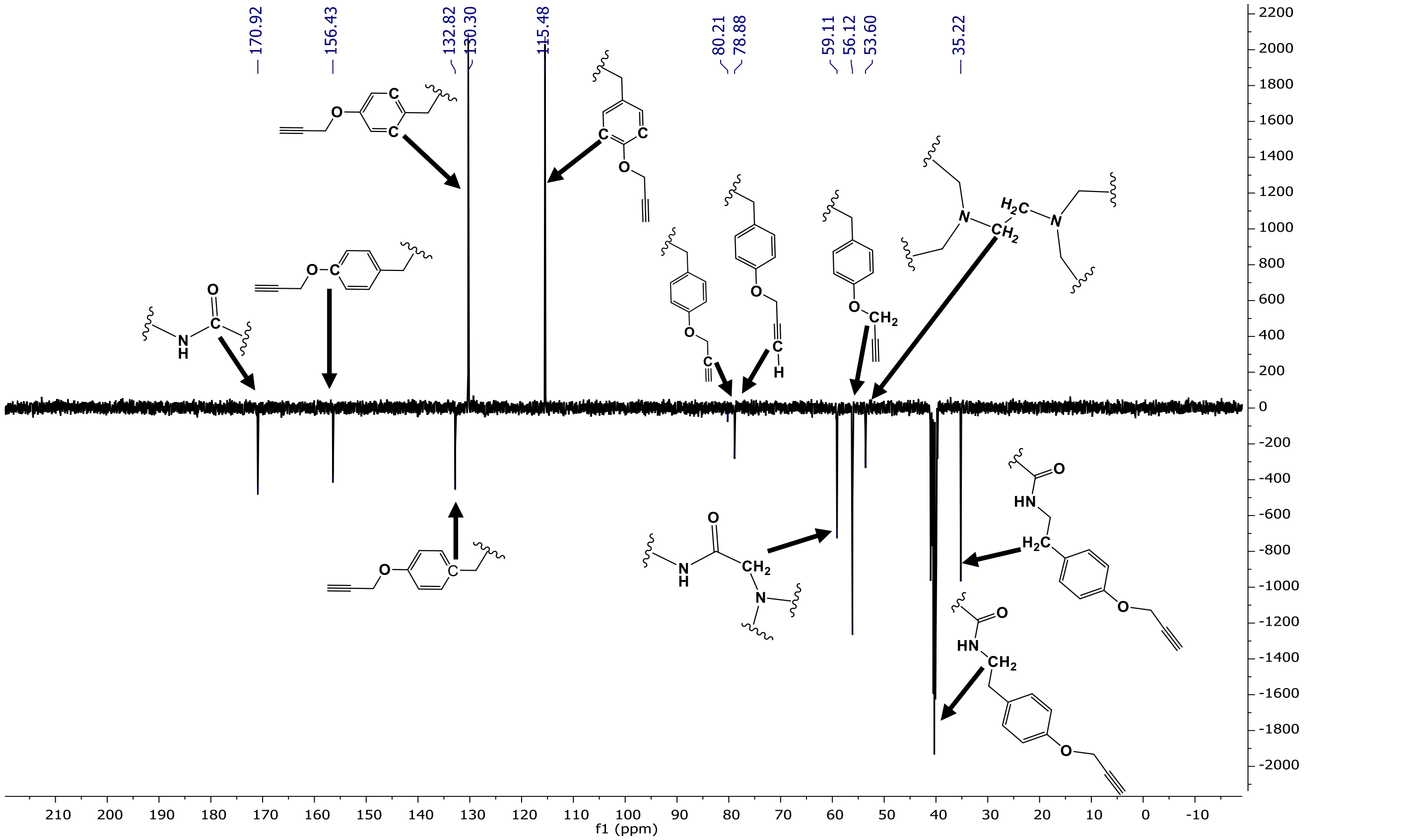

Figure S18. ^{13}C-NMR spectrum of tetrasubstituted EDTA G0-alkyne.

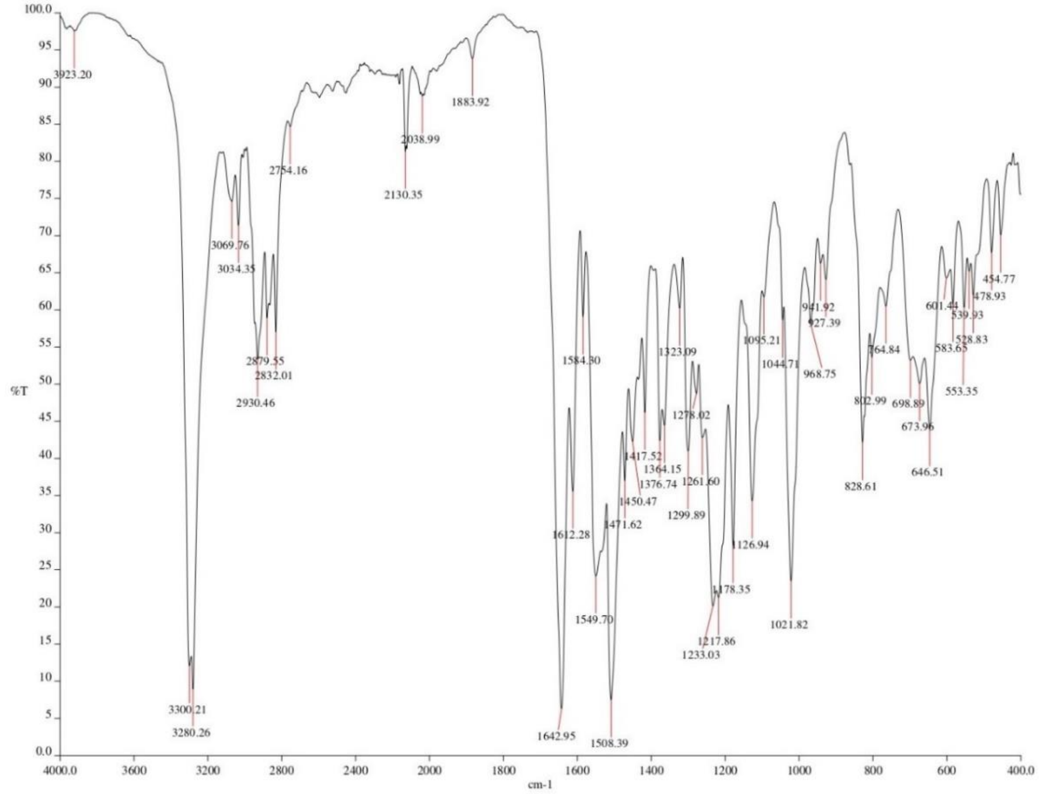

Figure S19. IR spectrum of tetrasubstituted EDTA G0-alkyne.

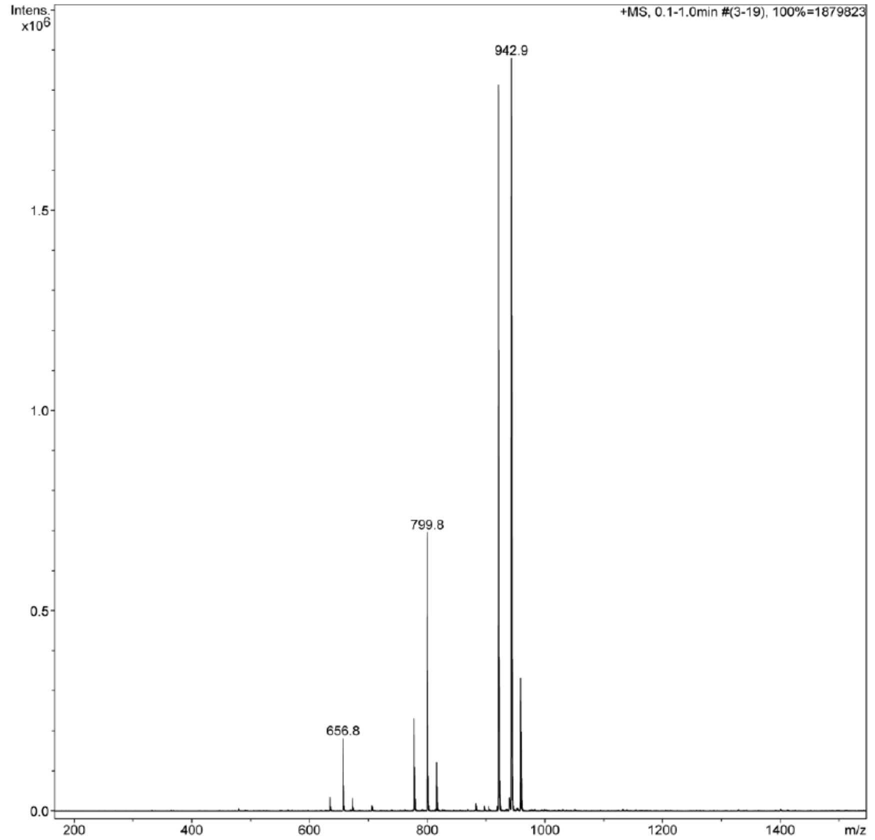

Figure S20. ESI spectrum of tetrasubstituted EDTA G0-alkyne.

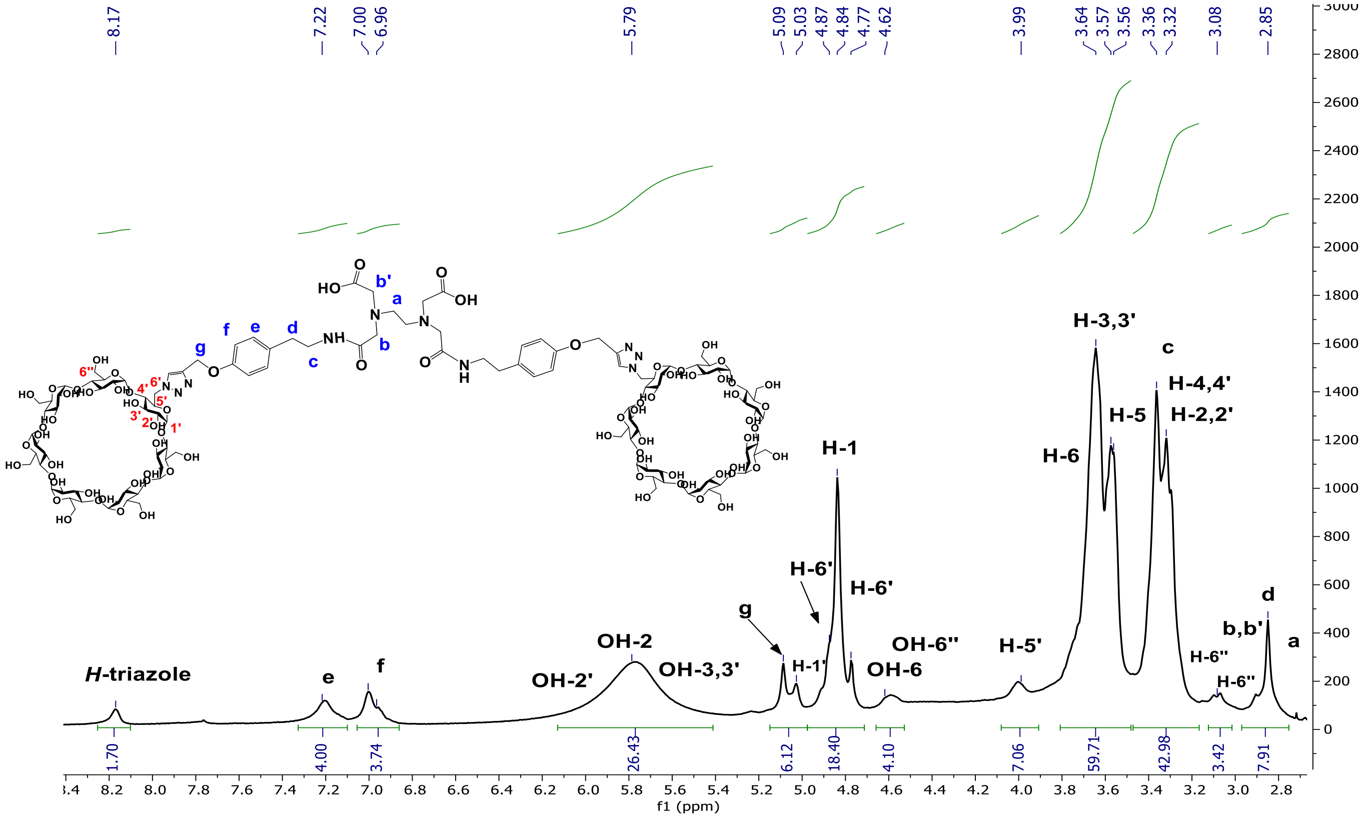

Figure S21. ^{1}H-NMR spectrum of dendritic EDTA di-βCD.

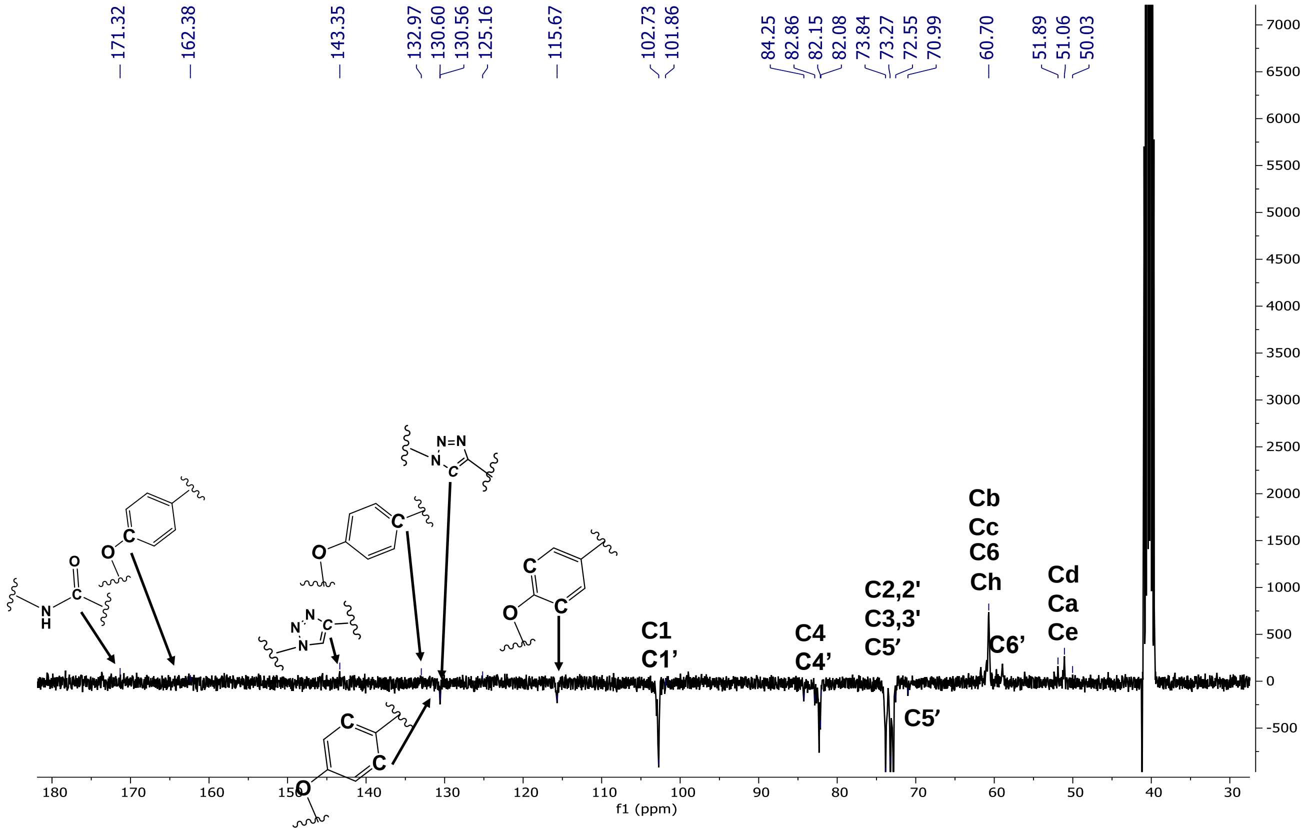

Figure S22. ^{13}C-NMR spectrum of dendritic EDTA di-βCD.

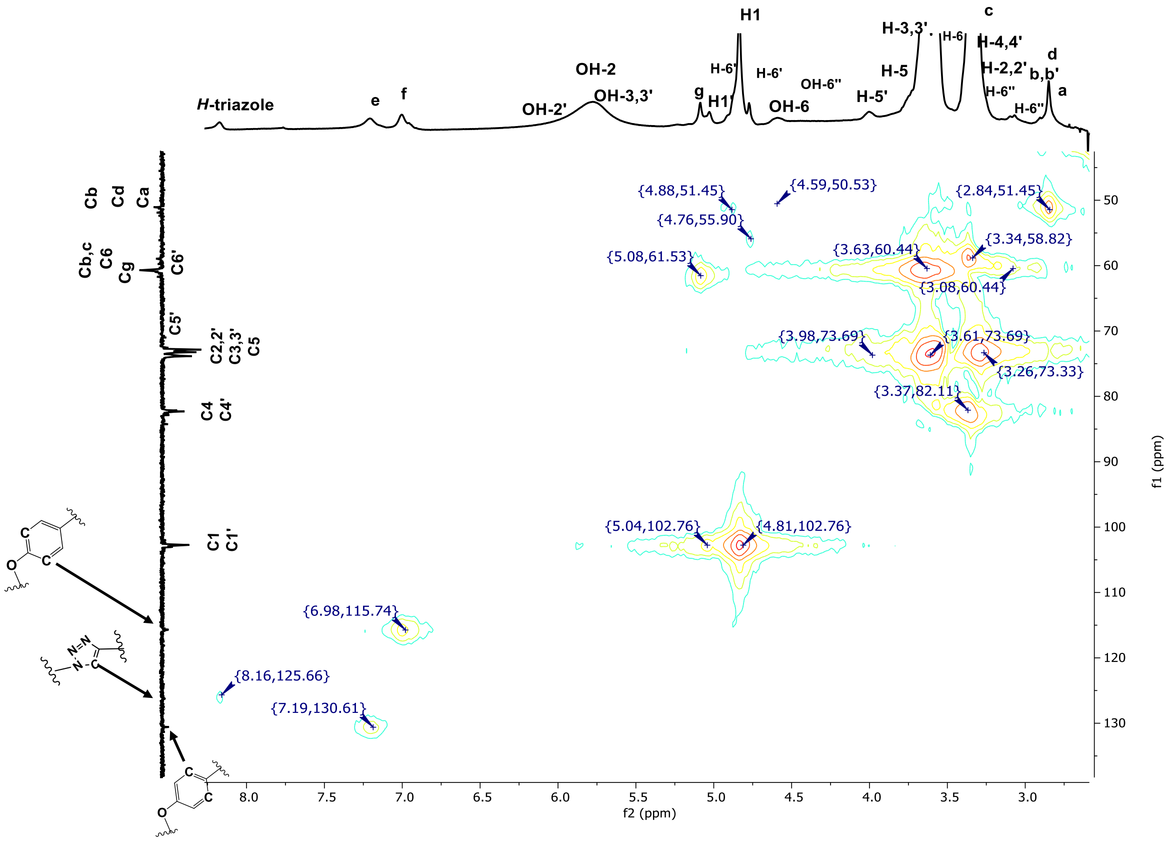

Figure S23. 2D NMR HMQC spectrum of dendritic EDTA di-βCD.

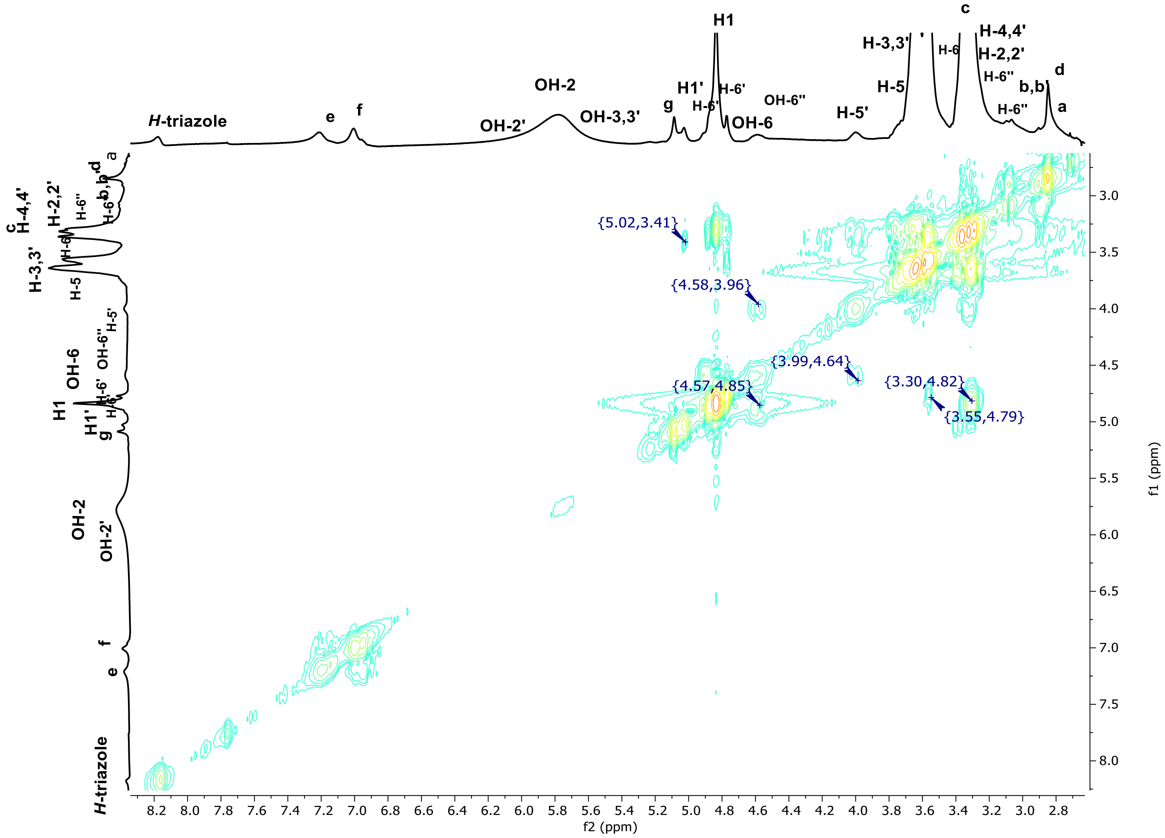

Figure S24. 2D NMR COSY spectrum of dendritic EDTA di-βCD.

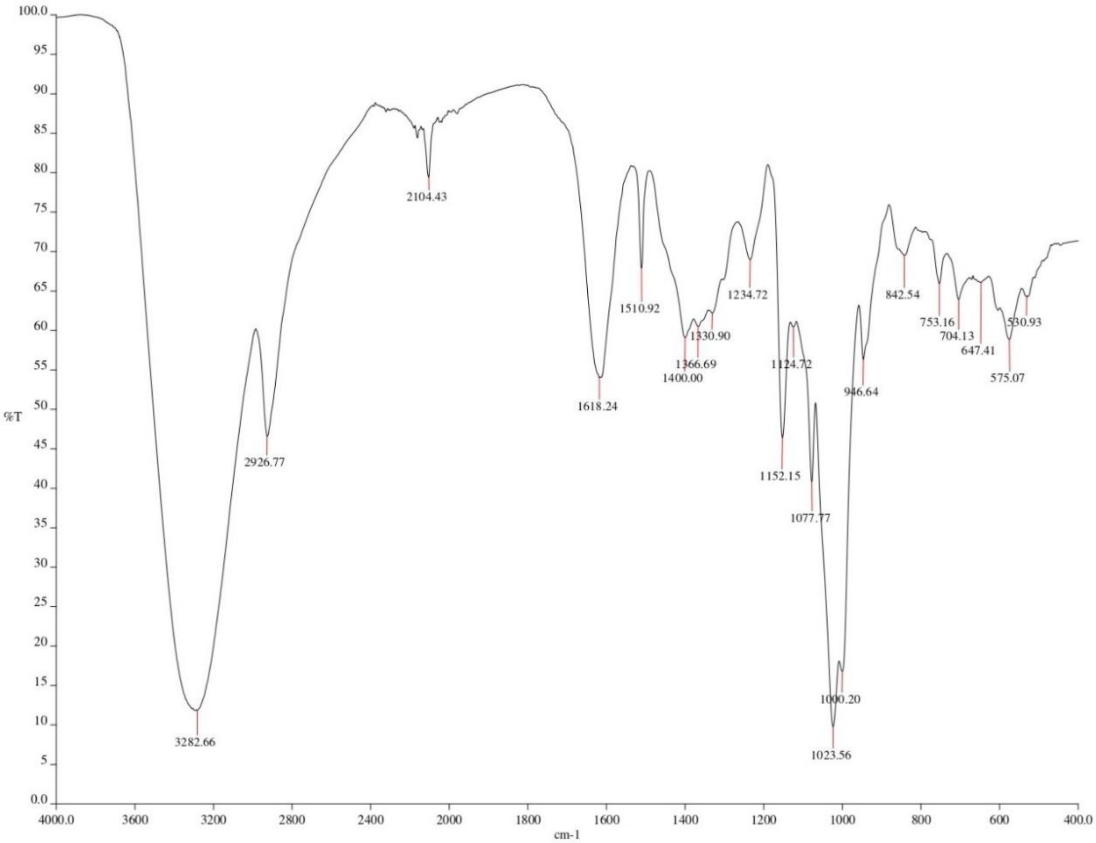

Figure S25. IR spectrum of dendritic EDTA di-βCD.

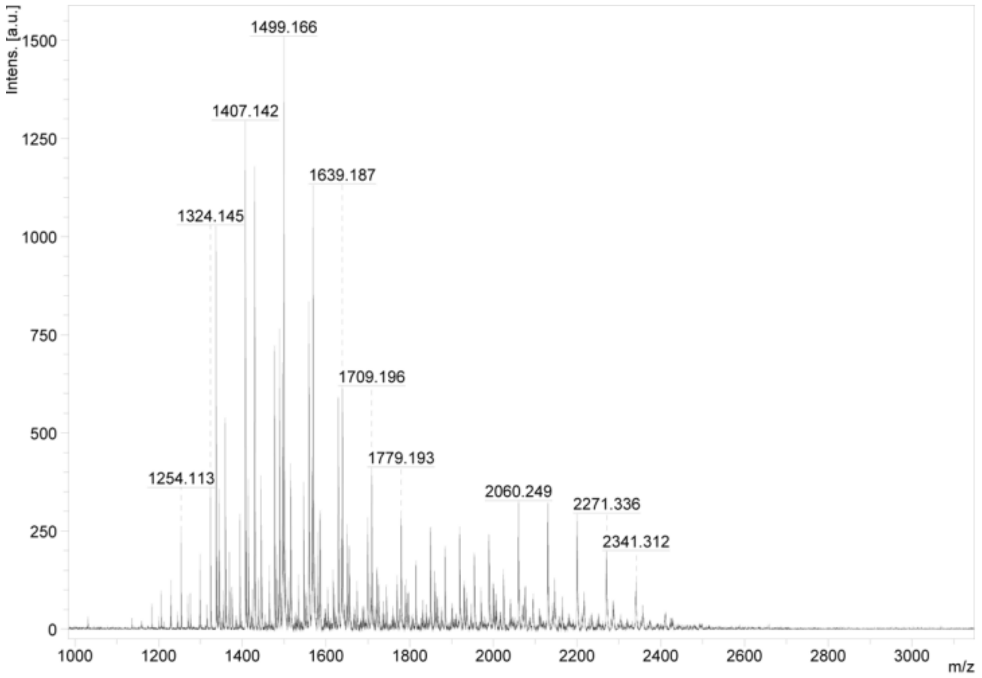

Figure S26. ESI-TOF spectrum of dendritic EDTA di-βCD.

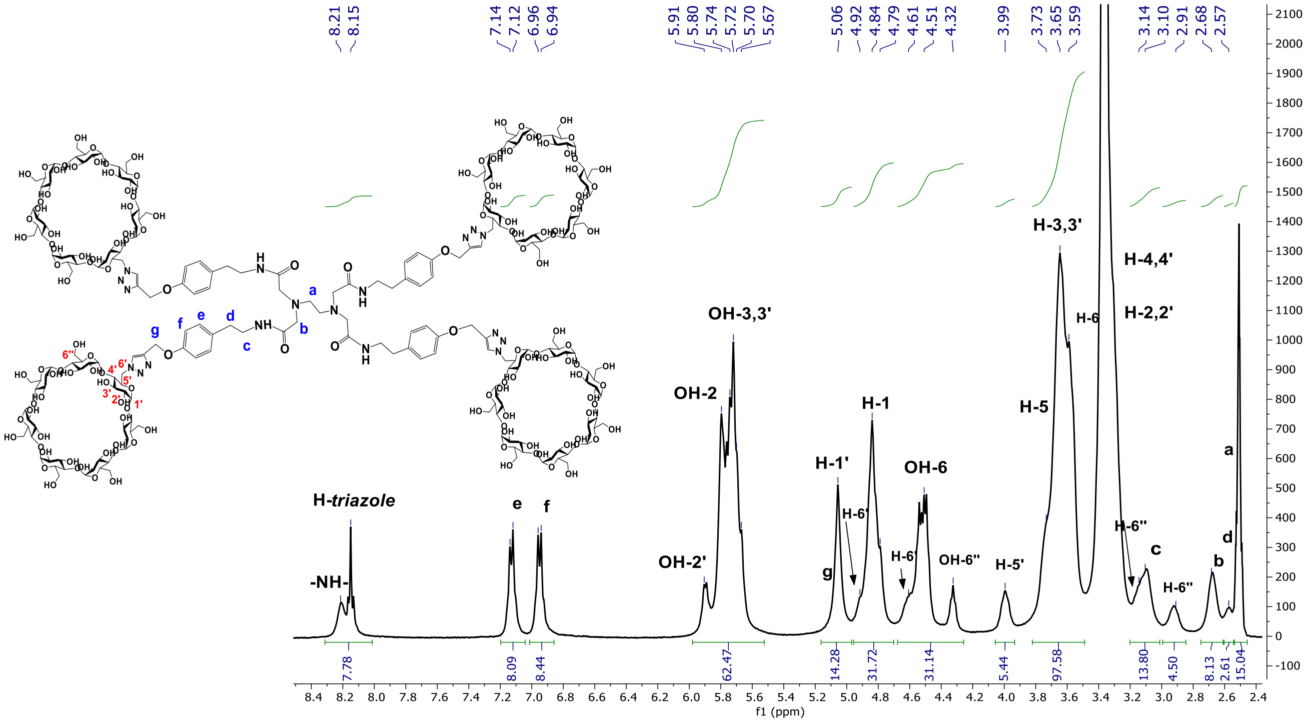

Figure S27. ^{1}H-NMR spectrum of EDTA G0-βCD dendrimer.

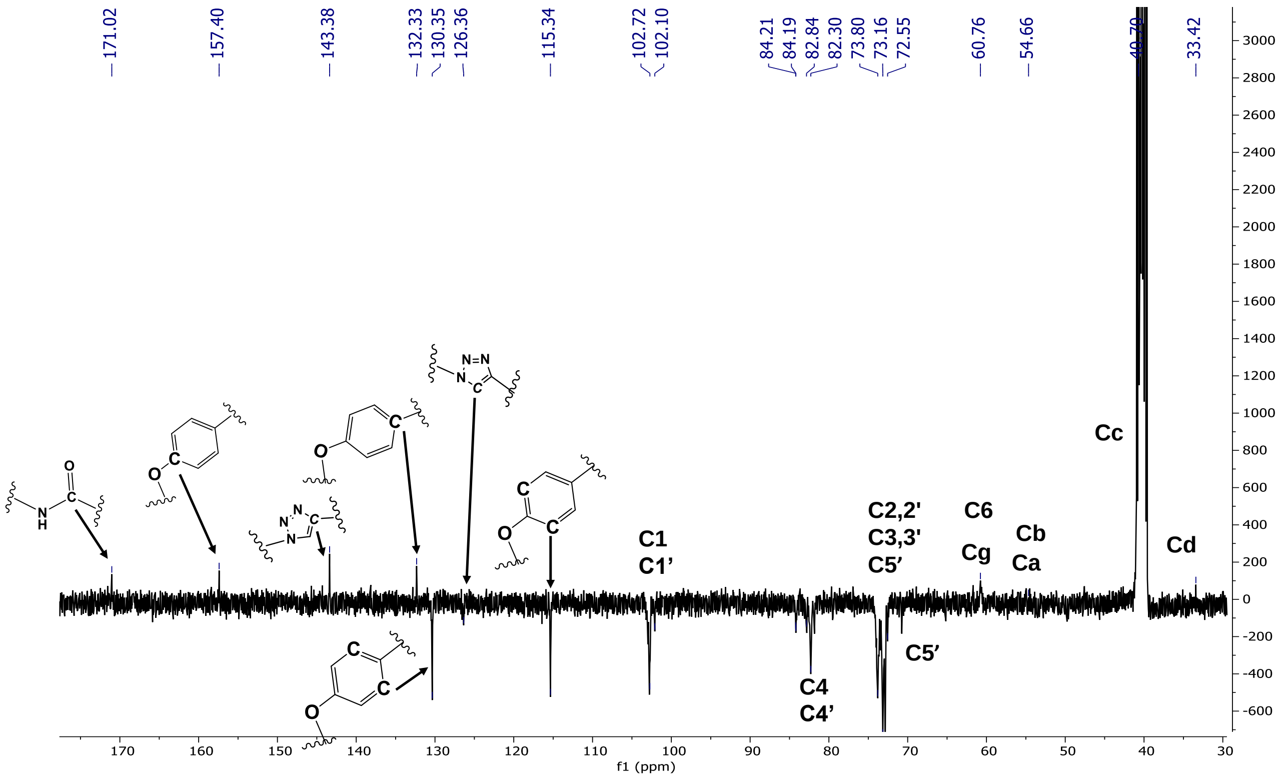

Figure S28. ^{13}C-NMR spectrum of EDTA G0-βCD dendrimer.

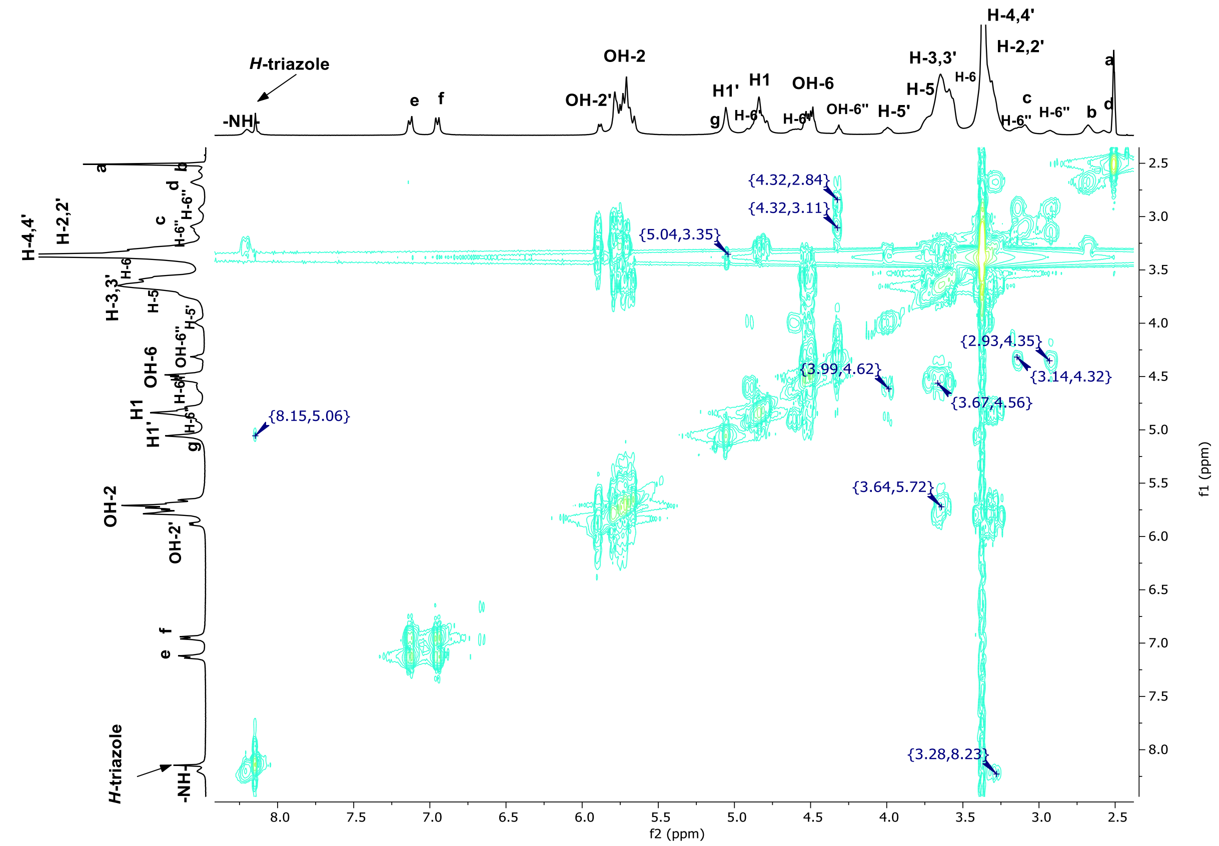

Figure S29. 2D NMR COSY spectrum of EDTA G0-βCD dendrimer.

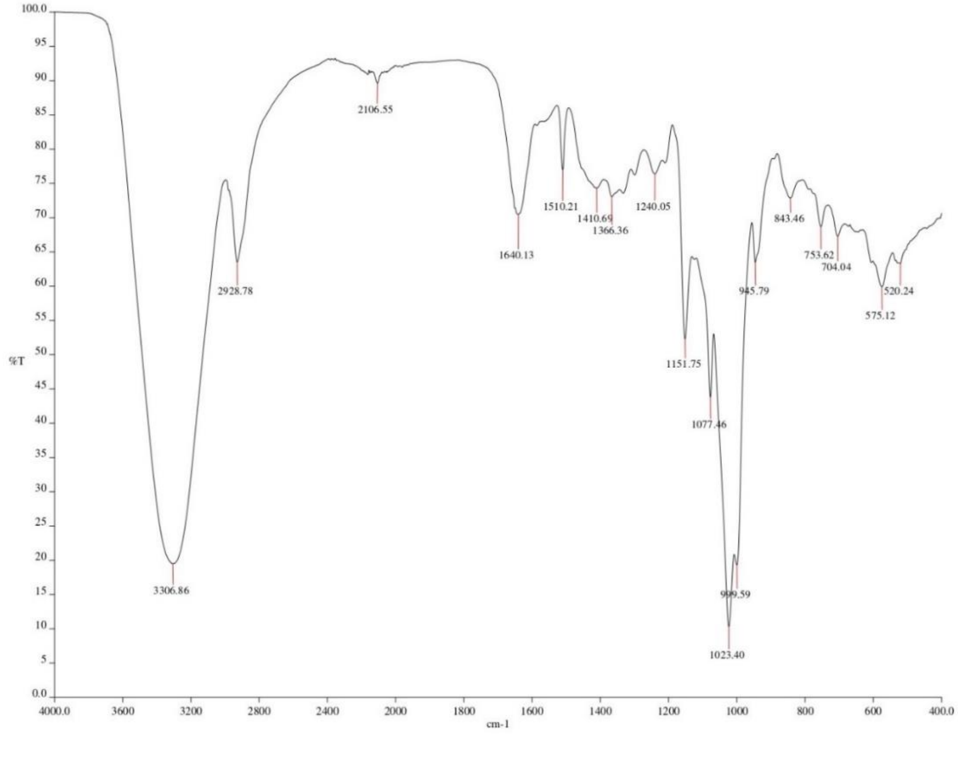

Figure S30. IR spectrum of EDTA G0-βCD dendrimer.

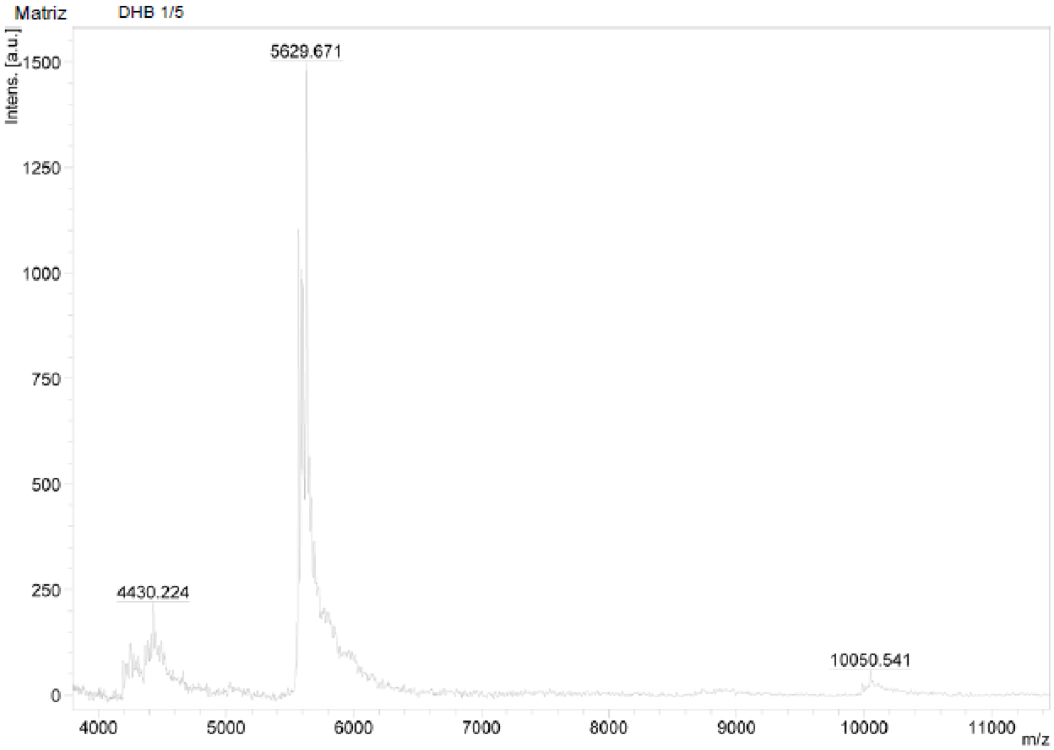

Figure S31. MALDI-TOF spectrum of EDTA G0-βCD dendrimer.
